# Calcium signaling induced by 15-deoxy-prostamide-J_2_ promotes cell death by activating PERK, IP3R, and the mitochondrial permeability transition pore

**DOI:** 10.18632/oncotarget.28334

**Published:** 2022-12-29

**Authors:** Daniel A. Ladin, Margaret M. Nelson, Estefani Cota, Catherine Colonna, Colin Burns, Jacques Robidoux, Kelsey H. Fisher-Wellman, Rukiyah Van Dross-Anderson

**Affiliations:** ^1^Medical Doctor Program, Brody School of Medicine, East Carolina University, Greenville, NC 27858, USA; ^2^East Carolina Diabetes and Obesity Institute at East Carolina University, Greenville, NC 27834, USA; ^3^Department of Physiology, Brody School of Medicine, East Carolina University, Greenville, NC 27834, USA; ^4^Department of Pharmacology and Toxicology, Brody School of Medicine, East Carolina University, Greenville, NC 27834, USA; ^5^Department of Chemistry, East Carolina University, Greenville, NC 27858, USA; ^6^UNC Lineberger Comprehensive Cancer Center, University of North Carolina at Chapel Hill School of Medicine, Chapel Hill, NC 27514, USA; ^7^Center for Health Disparities, East Carolina University, Greenville, NC 27834, USA

**Keywords:** calcium, mitochondrial respiration, endoplasmic reticulum stress, cancer, prostamide

## Abstract

Melanoma is the deadliest form of skin cancer in the US. Although immunotherapeutic checkpoint inhibitors and small-molecule kinase inhibitors have dramatically increased the survival of patients with melanoma, new or optimized therapeutic approaches are still needed to improve outcomes. 15-deoxy-Δ^12,14^-prostamide J_2_ (15d-PMJ_2_) is an investigational small-molecule that induces ER stress-mediated apoptosis selectively in tumor cells. Additionally, 15d-PMJ2 reduces melanoma growth *in vivo*. To assess the chemotherapeutic potential of 15d-PMJ_2_, the current study sought to uncover molecular pathways by which 15d-PMJ_2_ exerts its antitumor activity. B16F10 melanoma and JWF2 squamous cell carcinoma cell lines were cultured in the presence of pharmacological agents that prevent ER or oxidative stress as well as Ca^2+^ channel blockers to identify mechanisms of 15d-PMJ_2_ cell death. Our data demonstrated the ER stress protein, PERK, was required for 15d-PMJ_2_-induced death. PERK activation triggered the release of ER-resident Ca^2+^ through an IP_3_R sensitive pathway. Increased calcium mobilization led to mitochondrial Ca^2+^ overload followed by mitochondrial permeability transition pore (mPTP) opening and the deterioration of mitochondrial respiration. Finally, we show the electrophilic double bond located within the cyclopentenone ring of 15d-PMJ_2_ was required for its activity. The present study identifies PERK/IP3R/mPTP signaling as a mechanism of 15d-PMJ_2_ antitumor activity.

## INTRODUCTION

Melanoma, which originates in melanocytes of the epidermis, is the most aggressive and deadly form of skin cancer. It is estimated that approximately 100,000 new cases of melanoma will be diagnosed, and more than 7,000 individuals will succumb to the disease in 2022 [1]. Mortality associated with melanoma is low in early-stages however, the prognosis for late-stage disease is poor. In these advanced-stage patients, the use of immunotherapeutic checkpoint inhibitors has improved clinical outcomes. In addition, melanoma is highly responsive to small molecule inhibitors of MAPK pathway proteins including, BRAF and MEKK. Unfortunately, resistance to MAPK pathway inhibitors develops rapidly and checkpoint inhibitors are only effective in a fraction of patients with melanoma. Therefore, effective antineoplastic agents that can be administered with existing treatments or as monotherapies are needed to obtain greater improvement in patient survival.

Different studies have affirmed the efficacy of endoplasmic reticulum (ER) stress inducing agents against cancer [2, 3]. ER stress is a process that occurs when the protein folding efficiency in cells is compromised, causing an accumulation of unfolded proteins, and eliciting what is known as the unfolded protein response (UPR) [4, 5]. The presence of unfolded proteins in the ER causes the protein folding chaperone, GRP78 (BiP), to liberate three ER-localized UPR sensors, PERK, ATF6, and IRE1 to propagate their signal transduction cascades [6]. Activation of these UPR sensors blocks new protein synthesis, facilitates protein folding, and stimulates protein degradation in the ER to restore ER homeostasis and promote cell survival. However, in the presence of insurmountable ER stress, UPR sensor activation upregulates C/EBP homologous protein (CHOP10)/growth arrest and DNA damage inducible 153 (GADD153), a transcription factor that triggers apoptosis by modulating the expression of proapoptotic/antiapoptotic proteins including Bcl-2, death receptor 5 (DR5) and endoplasmic reticulum oxioreductase-1 (ERO1α) [5–9].

In addition to its role in protein folding, the ER, as well as the mitochondria serve as major Ca^2+^ reservoirs within the cell. In the ER, Ca^2+^ gradients needed for oxidative protein folding are established by the sarco/endoplasmic reticulum Ca^2+^-ATPase (SERCA) pump while excess calcium is released from the ER by the ryanodine (RyR) and inositol 1,4,5-triphosphate receptors (IP3R). In the mitochondria, Ca^2+^ that is required for cellular metabolism and ATP production is taken up into the outer mitochondrial membrane through the voltage-dependent anion-selective channel (VDAC) and into the mitochondrial matrix through the mitochondrial calcium uniporter (MCU). Slow discharge of Ca^2+^ from the mitochondria occurs through the VDAC, while large bursts of Ca^2+^ can exit during apoptosis through the mitochondrial permeability transition pore (mPTP). Under normal circumstances, IP_3_R-mediated Ca^2+^ transmission from the ER to the mitochondria supports ATP production [10]. However, during prolonged ER stress, sustained Ca^2+^ release may occur through interactions of IP3R with ER stress proteins including PERK, p20-BAP31, and Sig1R [7, 10, 11]. The sustained release of Ca^2+^ often leads to an increase in mitochondrial Ca^2+^ which promotes ROS generation and the sensitization of cyclophilin D, thereby contributing to the opening of the mPTP [12]. Stimulation of the mPTP results in the bolus release of cytochrome c into the cytoplasm, a crucial event in formation of the apoptosome, that causes irreversible induction of the intrinsic apoptotic pathway. Thus, Ca^2+^ transfer between the ER and mitochondria plays a vital role in regulating ER stress mediated apoptotic cell death.

15-deoxy-Δ^12,14^-prostaglandin J_2_ is a molecule that elicits death in diverse cancer cell types by targeting different signaling pathways and cellular processes including ER stress, PPARγ, and ROS [13–17]. It has also been demonstrated that 15-deoxy-Δ^12,14^-prostaglandin J_2_ promotes cell death by disrupting mitochondrial function. Specifically, 15-deoxy-Δ^12,14^-prostaglandin J_2_ was reported to block mitochondrial respiration by inhibiting complex I of the electron transport chain [18]. In addition, mitochondrial membrane potential was reduced, and cytoplasmic cytochrome C was increased in cells treated with 15-deoxy-Δ^12,14^-prostaglandin J_2_ [19]. The lipid, 15-deoxy-Δ^12,14^-prostaglandin J_2_, is generated when arachidonic acid is metabolized by COX-2 to prostaglandin H_2_ (PGH_2_). Through a series of enzymatic and non-enzymatic reactions, PGH_2_ is converted to prostaglandins including prostaglandin E_2_ (PGE_2_), prostaglandin F_2α_, (PGF_2α_), prostaglandin D_2_ (PGD_2_), and the J-series prostaglandins (prostaglandin J_2_, Δ^12,14^-prostaglandin J_2_, and 15-deoxy-Δ^12,14^-prostaglandin J_2_) which are terminal products of PGD_2_. Previous research has determined that COX-2 also metabolizes endocannabinoids including 2-arachidonoyl-glycerol (2-AG) and arachidonoyl-ethanolamide (AEA) to E-, F-, and D-series prostaglandin-glycerols and prostaglandin-ethanolamides (prostamides; PM), respectively. According to our data, the administration of exogenous AEA to tumor cells that overexpressed COX-2 led to the production of 15-deoxy-Δ^12,14^-prostamide J_2_ (15d-PMJ_2_). Our *in vitro* and *in vivo* studies show that 15d-PMJ_2_ caused tumor cell death by activating the cytotoxic ER stress pathway. In addition, the parent molecules of 15d-PMJ_2_, PMD_2_ and AEA, induced apoptosis in skin cancer cells by an ER- and oxidative-stress dependent mechanism [20–22]. AEA has diverse effects on Ca^2+^ mobilization and mitochondrial function in different cell types [23, 24]. However, the impact of 15d-PMJ_2_ on Ca^2+^ flux and mitochondrial activity and the involvement of these processes in melanoma cell death have not been investigated. Hence, the goal of the current work was to identify molecular mechanisms of 15dPMJ_2_ action by determining the importance of Ca^2+^ signaling in ER stress-apoptosis and examining the effects of this molecule on mitochondrial bioenergetics and function. Determining mechanisms by which 15dPMJ_2_ exerts its cytotoxic effect will provide insight into its potential utility in the clinical setting.

## RESULTS

### 15d-PMJ_2_-induced apoptosis is mediated by PERK

**Figure 1:**
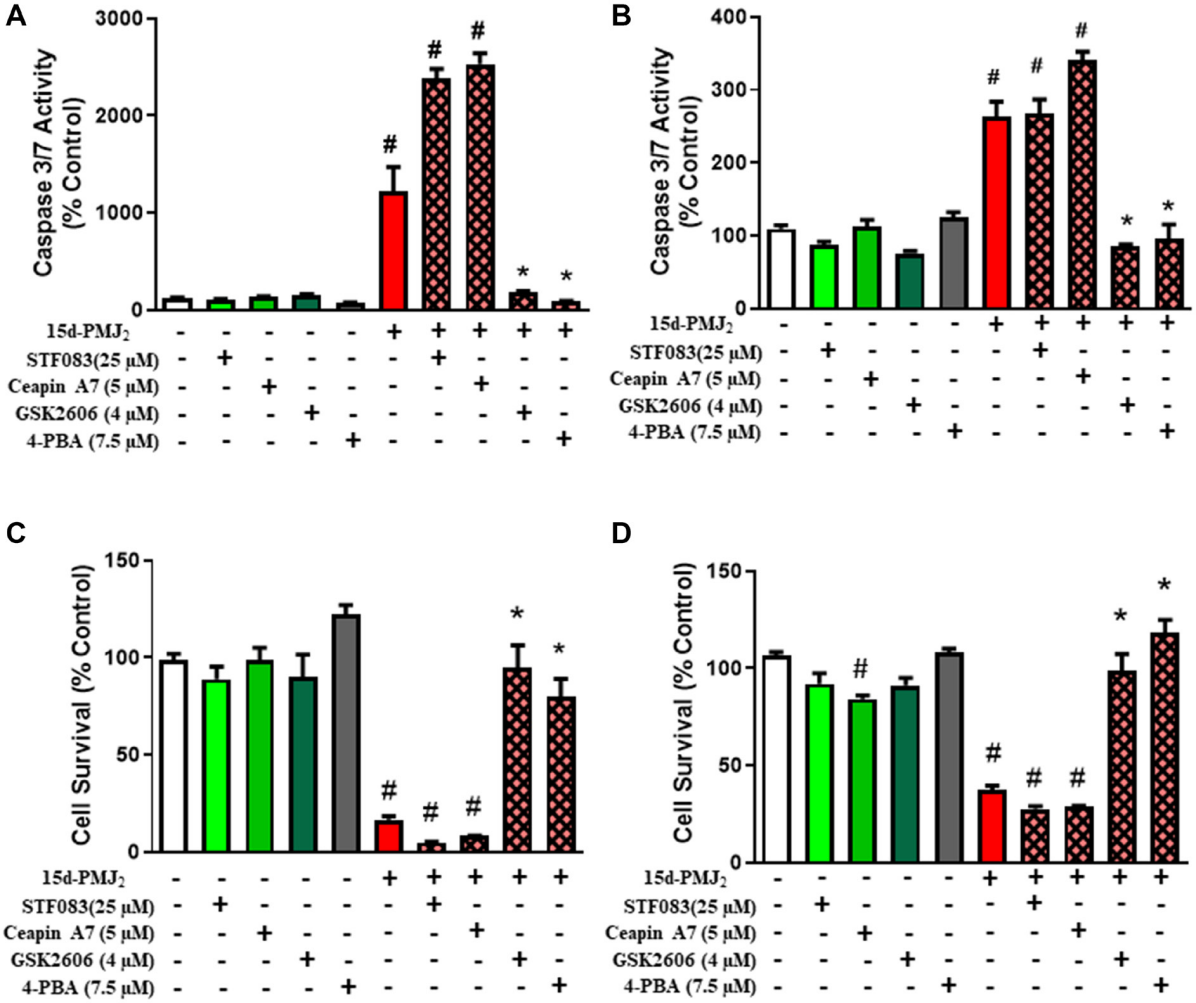
PERK activity is essential for 15d-PMJ_2_–induced apoptosis. JWF2 cutaneous squamous cell carcinoma and B16F10 melanoma cells were pretreated with STF038 (IRE inhibitor), Ceapin A7 (ATF6 inhibitor), GSK2606414 (PERK inhibitor), or 4-PBA (chemical chaperone) for 30 minutes and then the cells were treated with 5 μM 15d-PMJ_2_ or vehicle control (culture medium containing 0.1% DMSO) for different periods of time. (**A** and **B**) Caspase 3/7 activity was detected by utilizing Caspase Glo-3/7 reagents (Promega) after (A) 8 hours (JWF2) or (B) 16 hours (B16F10) of treatment with 15d-PMJ_2_ or vehicle. (**C** and **D**) Cell viability was evaluated by performing MTS experiments after (C) 12 hours (JWF2) or (D) 24 hours (B16F10) of treatment with 15d-PMJ_2_ or vehicle. The data are reported as the percent of untreated cells and are presented as the mean ± SEM of three independent experiments.^*^ *P <* 0.05, when comparing samples to 15d-PMJ_2_-treated cells, ^#^ *P <* 0.05, when comparing samples to vehicle-treated cells.

Previous work conducted by our group demonstrated that AEA and its metabolic product, 15d-PMJ_2_, caused ER stress-mediated cell death in tumorigenic keratinocytes and melanocytes [21, 22, 25]. Therefore, the current study sought to evaluate the three arms of the ER stress pathway, IRE1, ATF6 and PERK, to determine the predominant path by which 15d-PMJ_2_ increased apoptosis. To examine this question, JWF2 cutaneous squamous cell carcinoma (cSCC) and B16F10 melanoma cell lines were preincubated with the PERK inhibitor, GSK260414, the IRE1 inhibitor, STF083, or the ATF6 inhibitor, Ceapin A7, and the cells were then treated with 15d-PMJ_2_. The blockade of PERK in 15d-PMJ_2_-treated cells abolished the activity of caspase 3/7, which serves as an indicator of apoptosis (Figure 1A and 1B). In contrast, inhibition of IRE1 or ATF6, did not prevent caspase 3/7 activation (Figure 1A and 1B). Similarly, cell viability was significantly reduced by the suppression of PERK but not IRE1 or ATF6 (Figure 1C and 1D). Moreover, inhibition of the global unfolded protein response with the chemical chaperone, 4-PBA, abolished apoptosis and cell death that was caused by 15d-PMJ_2_ (Figure 1A–1D). These results demonstrate 15d-PMJ_2_-induced cell death is primarily regulated by the PERK pathway of the unfolded protein response.

### Oxidative-stress is not required for apoptotic cell death caused by 15d-PMJ_2_

**Figure 2:**
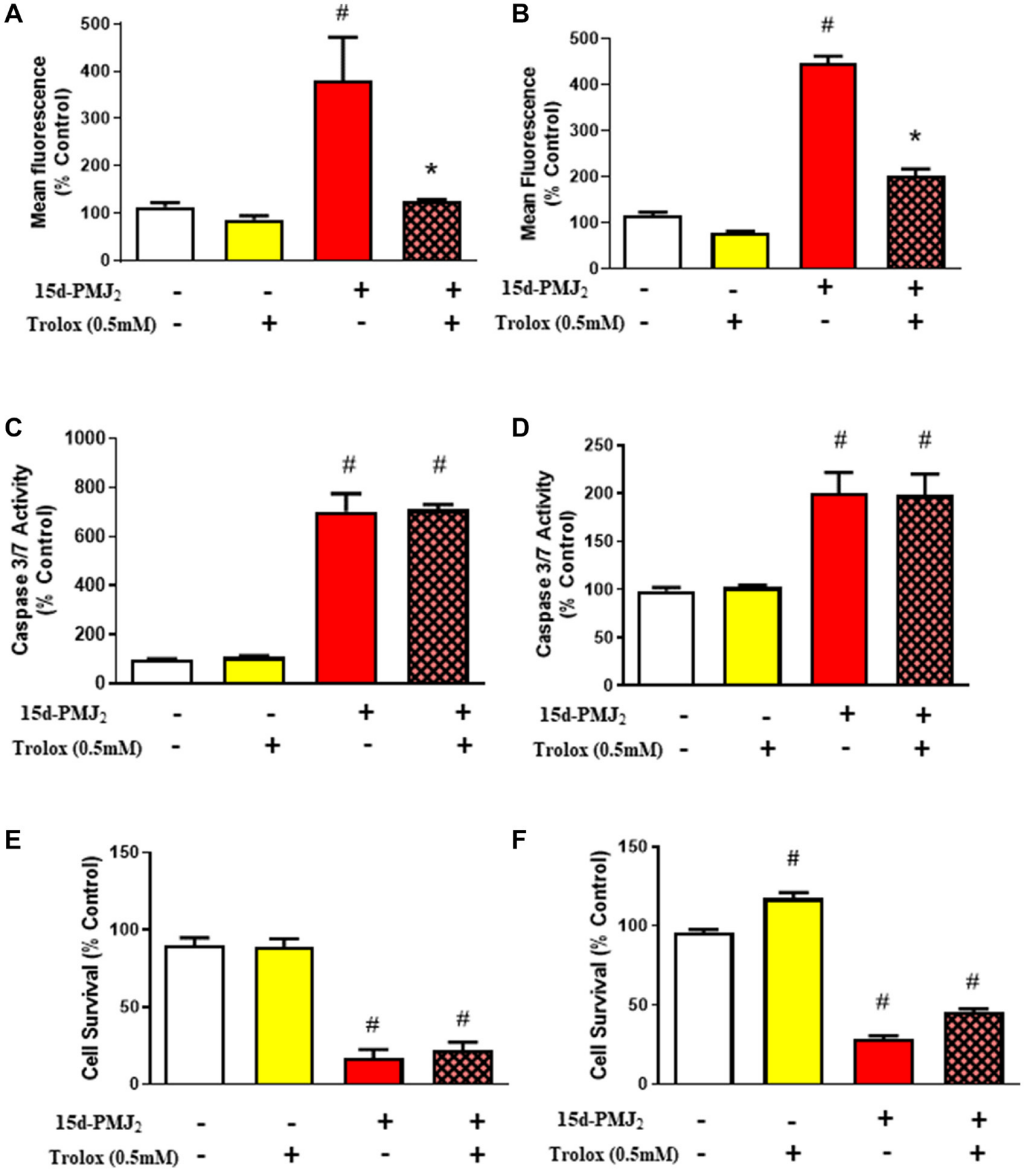
Oxidative stress is not required for 15d-PMJ_2_ induced cell death. JWF2 and B16F10 cells were pretreated with 0.5 mM Trolox for 30 minutes and then treated with 5 μM 15d-PMJ_2_ or vehicle for different periods of time. (**A** and **B**) Oxidative stress was detected by measuring CM-H_2_DCFDA fluorescence in (A) JWF2 or (B) B16F10 cells that were treated with 15d-PMJ_2_ or vehicle for 2 hours. (**C** and **D**) Apoptosis was detected by measuring Caspase 3/7 activity in cells treated with 5 μM 15d-PMJ_2_ or vehicle for (C) 8 hours (JWF2) or (D) 16 hours (B16F10). (**E** and **F**) Cell viability was determined by performing MTS experiments after treating the cells with 5 μM 15d-PMJ_2_ or vehicle for (E) 12 hours (JWF2) or (F) 24 hours (B16F10). The results are reported as the percent of untreated cells. The data are presented as the mean ± SEM of three independent experiments. ^*^ *P <* 0.05, when comparing samples to 15d-PMJ_2_-treated cells, ^#^ *P <* 0.05, when comparing samples to vehicle-treated cells.

Multiple reports indicate arachidonic acid-derived J-series prostaglandins cause cell death through the generation of oxidative stress [26, 27]. To test whether 15d-PMJ_2_ creates an oxidative environment, we examined the ability of 15d-PMJ_2_ to stimulate oxidation of the redox sensitive probe, H_2_DCFDA (DCF) [28]. 15d-PMJ_2_ increased DCF fluorescence by roughly 380% and 450% in JWF2 and B16F10 cells, respectively (Figure 2A and 2B). This phenomenon was reversed in cells that were pretreated with Trolox, a water-soluble vitamin E analog that exhibits antioxidant activity by neutralizing free radicals and lipid peroxidation [29]. To determine if oxidative stress plays a role in 15d-PMJ_2_-induced cell death, JWF2 and B16F10 cells were pretreated with Trolox and then treated with 15d-PMJ_2_. Interestingly, Trolox did not suppress caspase 3/7 activity (Figure 2C and 2D) or tumor cell death (Figure 2E and 2F) in either of the skin cancer-derived cell lines. These results indicate that although 15d-PMJ_2_ generates radical formation or lipid peroxidation, these oxidative molecules are not required for apoptosis or cell death in skin cancer cells.

### Activation of Ca^2+^ channels by 15d-PMJ_2_ is downstream of ER stress and required for cell death

**Figure 3:**
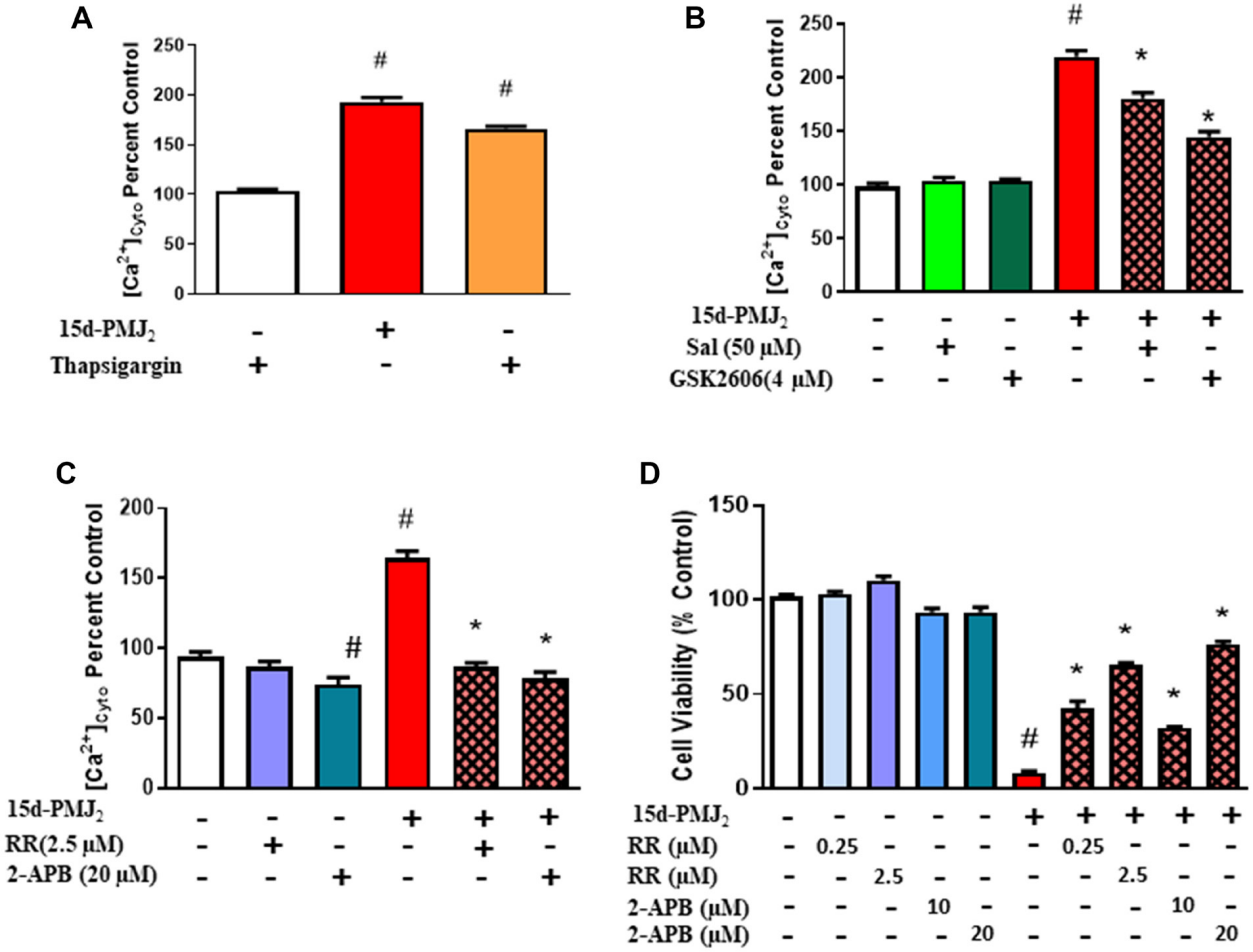
Activation of Ca^2+^ channels by 15d-PMJ_2_ is mediated by ER-stress and required for cell death. (**A**) B16F10 cells were treated with 5 μM 15d-PMJ_2_ or vehicle. As a positive control, the cells were also treated with thapsigargin (10 μM), a prototype ER stress inducer that increases cytoplasmic calcium levels by inhibiting the SERCA pump. Cytoplasmic Ca^2+^ was measured by performing assays with Fluor-4 NW. (**B**) B16F10 cells were pretreated for 30 minutes with the ER stress inhibitors, salubrinal (50 μM) and GSK2606414 (4 μM). The cells were treated with 5 μM 15d-PMJ_2_ or vehicle for 1 hour and cytoplasmic Ca^2+^ was detected. (**C**) B16F10 cells were pretreated for 1 hour with the calcium channel blockers, ruthenium red (RR, 2.5 μM) and 2-APB (20 μM). The cells were then treated with 5 μM 15d-PMJ_2_ or vehicle for 1 hour and cytoplasmic Ca^2+^ was measured. (**D**) Cells were pretreated with RR (0.25 or 2.5 μM) or 2-APB (10 or 20 μM) for 1 hour followed by cell treatment with 5 μM 15d-PMJ_2_ or vehicle for 24 hours. Cell viability was determined by conducting MTS experiments. The data are presented as the mean ± SEM of three independent experiments. ^*^ *P <* 0.05, when comparing samples to 15d-PMJ_2_-treated cells, ^#^ *P <* 0.05, when comparing samples to vehicle-treated cells.

Different studies demonstrate that under conditions of ER stress, Ca^2+^ efflux from the ER into cytosol or mitochondria facilitates apoptosis [30, 31]. In addition, Ca^2+^ efflux from the ER can stimulate apoptosis through activation of ER stress pathway proteins including PERK [32, 33]. As such, we sought to determine if ER stress induced by 15d-PMJ2 causes an increase in cytosolic Ca^2+^ that is required to trigger an apoptotic response. To measure cytosolic calcium levels, B16F10 melanoma cells were loaded with the cytosolic calcium probe, Fluo-4-NW, and then the cells were treated with 15d-PMJ2. 15d-PMJ_2_ caused a significant increase in cytosolic calcium compared to control (Figure 3A). To examine the role of ER stress in cytoplasmic Ca^2+^ localization, the activity of PERK was blocked with salubrinal, an indirect, and GSK2606414, a direct inhibitor of PERK. Both PERK inhibitors significantly lowered 15d-PMJ_2_-induced cytosolic Ca^2+^ compared to cells treated with 15d-PMJ2 alone, suggesting Ca^2+^ transit to the cytoplasm is facilitated by PERK-mediated ER stress (Figure 3B). ER stress proteins, including PERK, regulate the release of ER-resident Ca^2+^ through their interaction with IP3R [34]. Therefore, we evaluated whether IP_3_R was responsible for the increase in cytosolic calcium by using the IP3R inhibitor, 2-aminoethoxydiphenyl borate (2-APB), and the non-selective calcium channel blocker, ruthenium red (RR). 2-APB and RR caused a nearly complete reversal of the increase in cytosolic Ca^2+^ initiated by 15d-PMJ2 suggesting that the Ca^2+^ originates from IP3R-regulated, ER stores (Figure 3C). Next, we investigated whether the increase in cytosolic Ca^2+^ was crucial for cell death. The calcium channel blockers, 2-APB and RR reversed the cytotoxicity of 15d-PMJ2 in B16F10 cells (Figure 3D). A similar outcome was observed in JWF2 cSCC cells (Supplementary Figure 1A–1D). These data indicate 15d-PMJ_2_-mediated activation of PERK leads to an increase in cytosolic calcium via IP_3_R.

### 15d-PMJ_2_ increases mitochondrial calcium flux and membrane permeability through PERK activation

**Figure 4:**
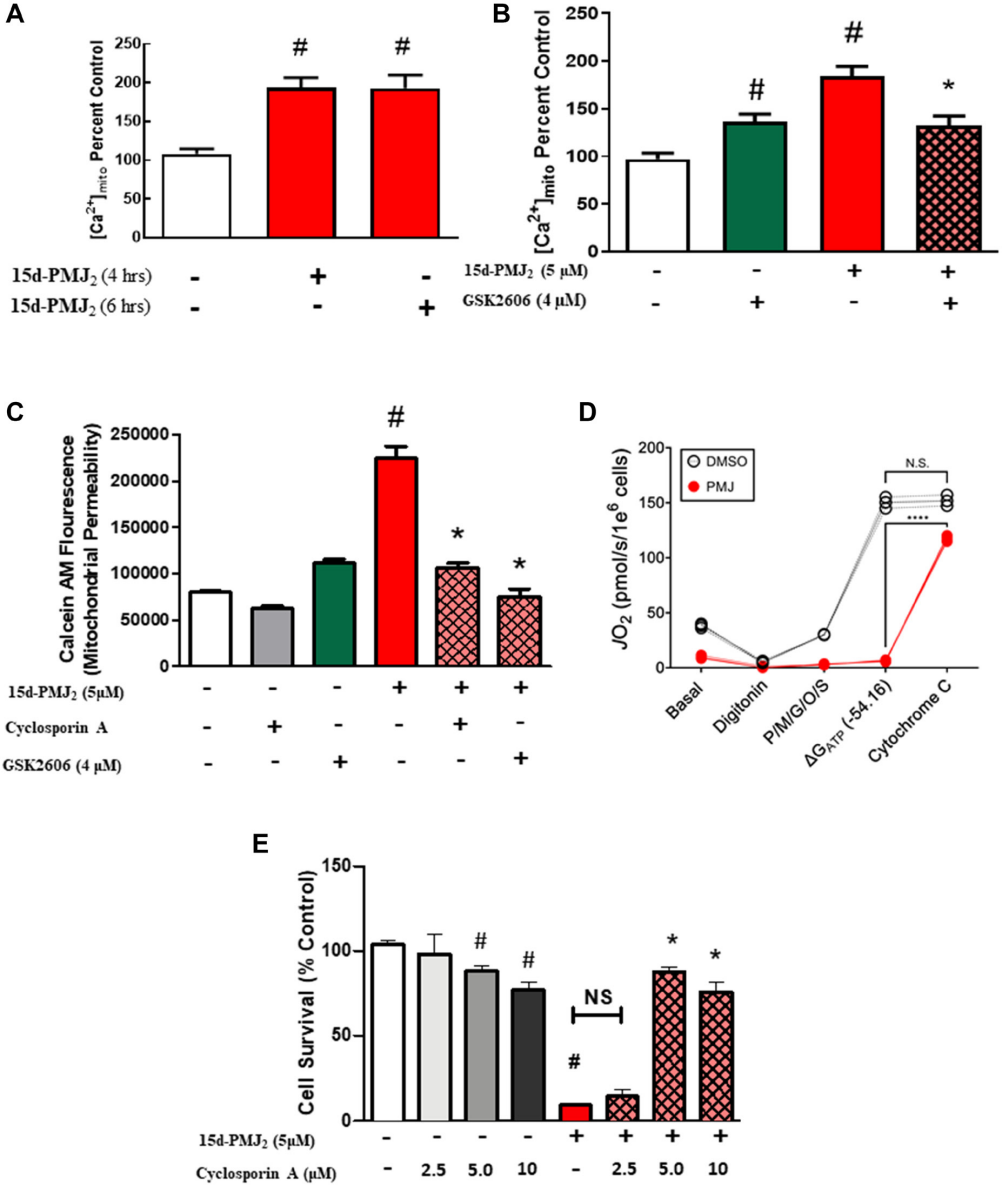
15d-PMJ_2_-mediated ER-stress increases mitochondrial calcium flux and membrane permeability. (**A**) B16F10 cells were treated with vehicle or 15d-PMJ_2_ (5 μM) for 4 or 6 hours and mitochondrial Ca^2+^ levels were measured by detecting Rhod-2 fluorescence by flow cytometric analysis. (**B**) B16F10 cells were pretreated for 30 minutes with the ER-stress inhibitor, GSK2606414 (4 μM), treated with 5 μM 15dPMJ_2_ or vehicle for 6 hours, and mitochondrial Ca^2+^ levels were detected. (**C**) B16F10 cells were pretreated with the mPTP inhibitor, cyclosporin A (1 hour), or the ER stress inhibitor, GSK2606414 (30 minutes), and then the cells were treated with 15d-PMJ_2_ or vehicle for 6 hours. Cell fluorescence was then quenched with CoCl_2_ and assayed with Calcein-AM by flow cytometry. (**D**) B16F10 cells were treated for 12 hours with 15d-PMJ_2_ (or vehicle), and mitochondrial respiration was assessed in digitonin permeabilized cells after 12 hours. Following an assessment of basal respiration, cells were permeabilized with digitonin, and energized with pyruvate/malate/glutamate/octanoyl-carnitine/succinate (P/M/G/O/S). Respiration was stimulated with ATP free energy (ΔG_ATP_ −54.16 kJ/mol). Cytochrome C was then added to assess the permeability of the outer mitochondrial membrane. ^*^Different from the ΔG_ATP_ −54.16 kJ/mol condition (*N* = 3/group). (**E**) B16F10 cells were pretreated with cyclosporin A for 1 hour and then treated with 15d-PMJ_2_ for 24 hours. Cell viability was determined by conducting MTS experiments. Data represent mean ± SEM of three independent experiments and are expressed as (A–C, E) percent of untreated group or (D) pmol/s/million cells. ^#^ *P <* 0.05, as compared to vehicle, ^*^ *P <* 0.05, as compared to 15d-PMJ_2_.

Elevated mitochondrial calcium levels are known to contribute to the apoptotic cascade through opening of the mPTP which permits the release of cytochrome C [31]. As such, we investigated whether 15d-PMJ_2_ increased mitochondrial Ca^2+^ levels and mPTP opening and if this effect was regulated by ER stress. In B16F10 cells, 15d-PMJ_2_ caused an increase in the fluorescence of Rhod-2, a calcium probe that accumulates in the mitochondria (Figure 4A). Blockade of PERK with GSK2606414 in 15d-PMJ_2_-treated cells led to a significant reduction in mitochondrial calcium suggesting that PERK-dependent calcium release is essential for mitochondrial Ca^2+^ accumulation (Figure 4B). To determine if 15d-PMJ_2_ increases mPTP opening and potential mechanisms behind this effect, mitochondrial permeability was assayed by measuring Calcein-AM fluorescence in the presence of mitochondrial impermeable quencher, CoCl_2_. 15d-PMJ_2_ significantly increased opening of the mPTP and this effect was abolished by the inhibition of PERK (Figure 4C). We confirmed the impact of 15d-PMJ_2_ on mPTP opening by measuring mitochondrial respiration. Following an assessment of basal respiration, B16F10 cells were exposed to either vehicle or 15d-PMJ_2_ and then the cells were permeabilized with digitonin to allow direct access to the mitochondrial network. Respiration was then stimulated with saturating carbon substrate and ATP free energy. Despite robust respiration in the vehicle treated cells (Figure 4D), cells exposed to 15d-PMJ_2_ were refractory to the addition of both carbon substrate and ATP free energy (Figure 4D). However, upon the addition of exogenous cytochrome C, respiration in 15d-PMJ_2_ cells was restored nearly to the rate of vehicle treated cells, entirely consistent with loss of cytochrome C through mPTP (Figure 4D). Lastly, to evaluate whether mPTP opening was required for 15d-PMJ_2_-mediated cell death, cell viability was measured in melanoma cells pretreated with cyclosporin A, a known mPTP inhibitor. Cyclosporin A was found to abrogate 15d-PMJ_2_-mediated cell death (Figure 4E) demonstrating that induction of the mPTP was required for its cytotoxicity.

### Aberrant changes to mitochondrial respiration elicited by 15d-PMJ_2_ are caused by ER stress- and calcium-dependent mechanisms

**Figure 5:**
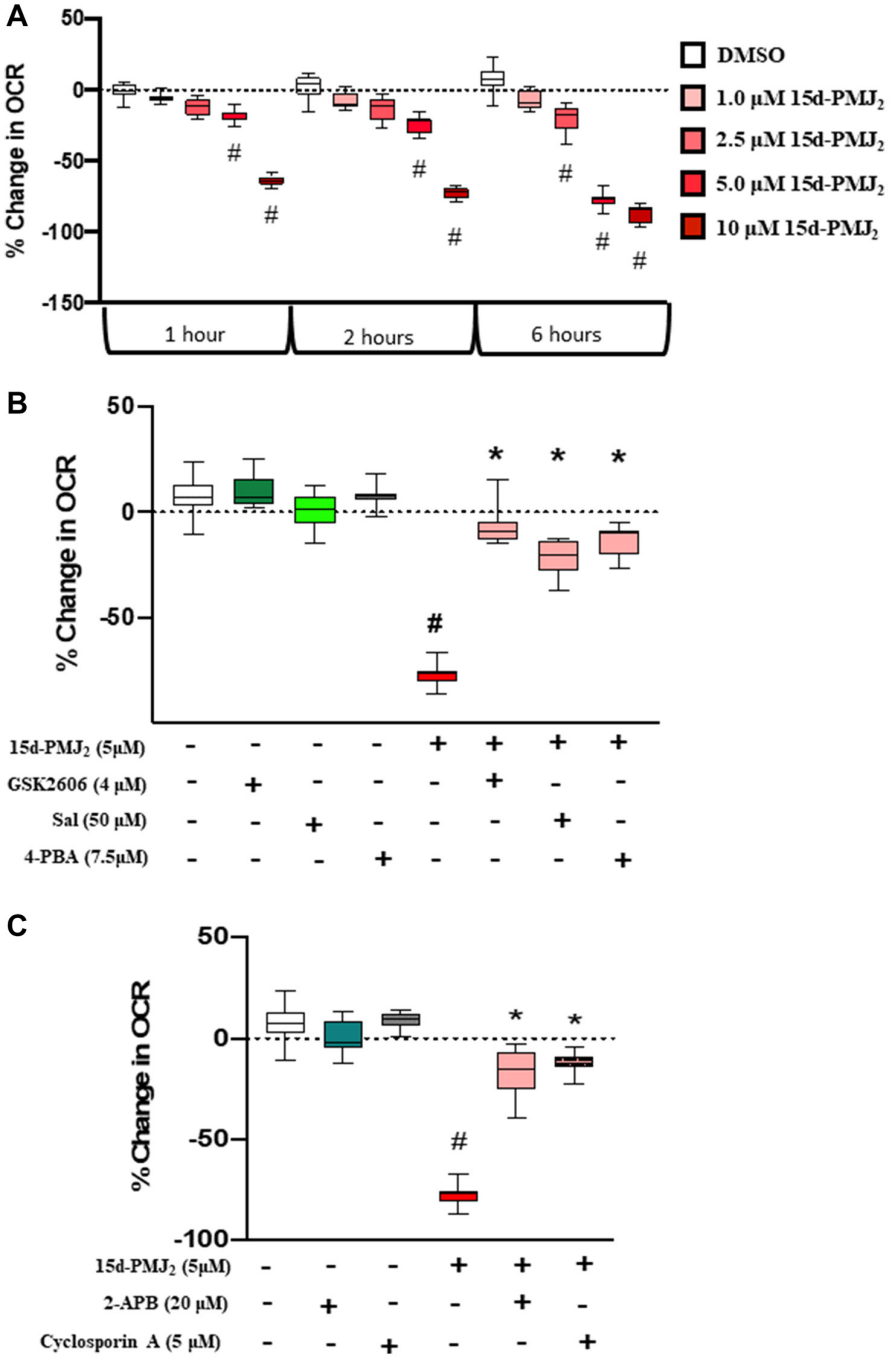
15d-PMJ_2_ decreases mitochondria respiration in a dose- and ER stress-dependent manner. (**A**) B16F10 cells were treated with vehicle or different concentrations of 15d-PMJ_2_. (**B**) B16F10 cells were pretreated with different inhibitors for 30 minutes followed by incubation with 15d-PMJ_2_. (**C**) B16F10 cells were pretreated with 2-APB or cyclosporin A for 1 hour and then treated with 15d-PMJ_2_. Mitochondrial oxygen consumption rate was measured using the Seahorse XFe96 Flux analyzer every 10 minutes for 6 hours. Data represent mean ± SEM of three independent experiments. ^#^ *P <* 0.05, as compared to vehicle, ^*^ *P <* 0.05, as compared to 15d-PMJ_2_.

Different studies have shown that J-series prostaglandins, such as15-deoxy-Δ^12,14^ prostaglandin J_2_, inhibit mitochondrial function by influencing mitochondrial respiration and inhibiting complex 1 of the electron transport chain [18, 35]. Our results indicate that the structurally similar molecule, 15d-PMJ_2_, suppresses cellular respiration via the mPTP in an ER stress/IP_3_R- dependent manner (Figure 4). Therefore, we characterized the impact of 15d-PMJ_2_ on mitochondrial bioenergetics and evaluated whether 15d-PMJ_2_-induced ER stress or calcium channel activity influences cellular respiration. In intact B16F10 cells, 15d-PMJ_2_ was found to significantly reduce cellular respiration in a concentration-dependent manner (Figure 5A). Consistent with these results, we found the suppression of cellular respiration by 15d-PMJ_2_ was abrogated in the presence of the ER stress inhibitors, 4-PBA, GSK2606414, and salubrinal (Figure 5B). Notably, inhibition of the mPTP and IP_3_R with cyclosporin A and 2-APB respectively, prevented the reduction in melanoma cell respiration by 15d-PMJ_2_ (Figure 5C). These data indicate that PERK, IP_3_R-mediated calcium flux, and the mPTP play a crucial role in the disruption of cellular respiration by 15d-PMJ_2_.

To determine if 15d-PMJ_2_ inhibits cellular respiration by directly targeting the mitochondrial metabolism machinery or by interacting with matrix enzymes, we assessed mitochondrial respiration and dehydrogenase activity in intact mitochondria isolated from B16F10 cells. Basal mitochondrial respiration, mitochondria energized with glutamate/malate (Supplementary Figure 2A), octanoyl-carnitine/malate (Supplementary Figure 2B), succinate/rotenone (Supplementary Figure 2C) and mitochondrial enzyme activity (Supplementary Figure 2D–2F) were evaluated in the presence of 15d-PMJ_2_. 15d-PMJ_2_ exhibited no significant impact on mitochondrial respiration or mitochondrial enzyme activity compared to control (Supplementary Figure 2A–2F). These results indicate that unlike chemically similar 15-deoxy-Δ^12,14^ prostaglandin J_2,_ 15d-PMJ_2_ does not directly influence mitochondrial bioenergetics or complex 1 activity.

### 15d-PMJ_2_ promotes apoptosis through its cyclopentenone double bond

**Figure 6:**
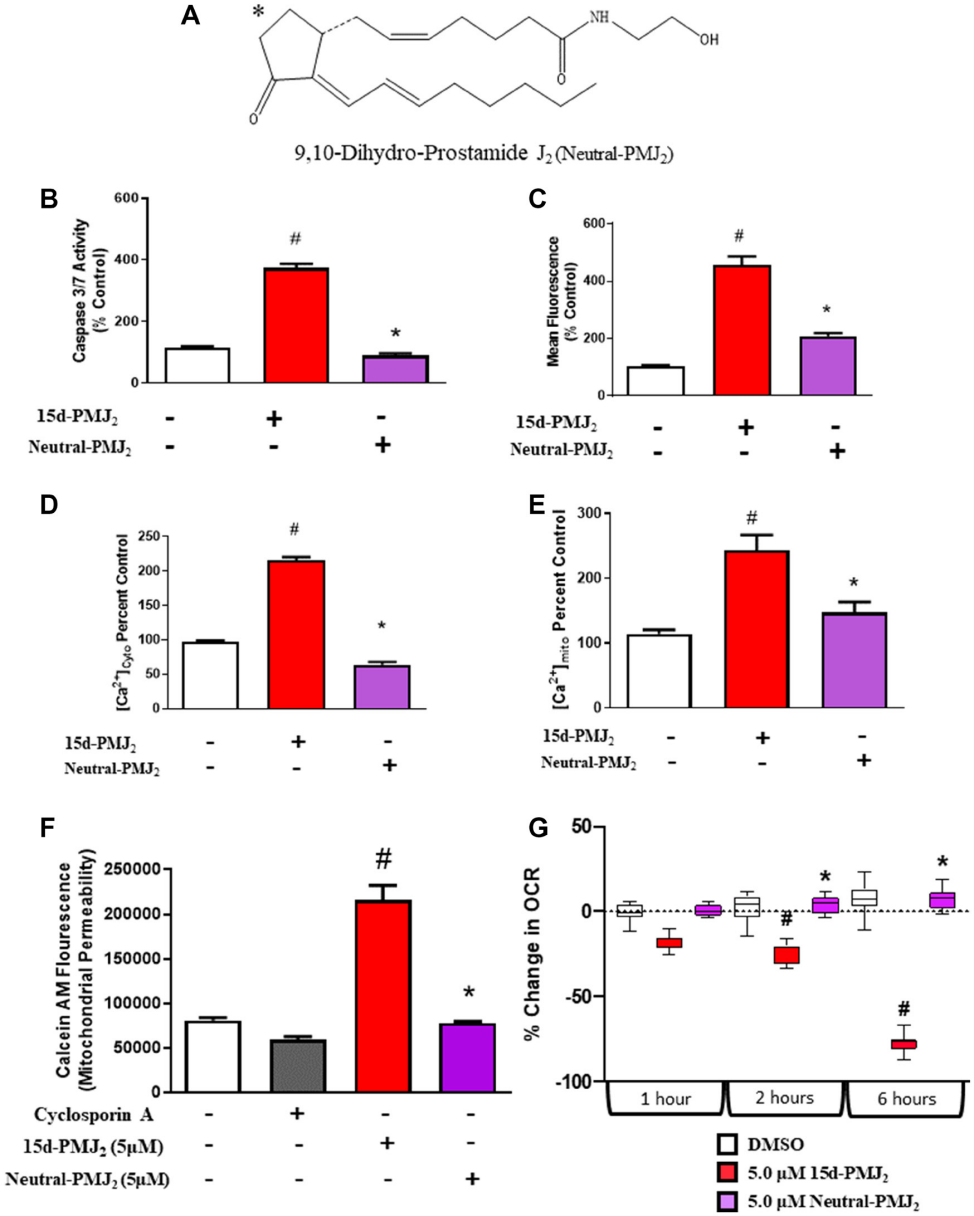
The activity of 15d-PMJ_2_ is regulated by its electrophilic double bond. (**A**) The structure of Neutral-15d-PMJ_2_ is presented. B16F10 cells were treated with 5 μM 15d-PMJ_2_, 5 μM Neutral-15d-PMJ_2_ or vehicle and then assayed for (**B**) Caspase-3/7 activity, (**C**) oxidative stress, (**D**) cytoplasmic Ca^2+^, (**E**) mitochondrial Ca^2+^, (**F**) mitochondrial permeability, and (**G**) mitochondrial oxygen consumption. Data represent mean ± SEM of three independent experiments and are expressed as percent of untreated group. ^#^ *P <* 0.05, as compared to vehicle, ^*^ *P <* 0.05, as compared to 15d-PMJ_2_.

The current work demonstrates 15d-PMJ2 causes tumor cell apoptosis via a PERK/IP_3_R/mPTP-dependent pathway. Furthermore, our lab has previously shown that 15d-PMJ_2_-induced ER stress and cell death are conferred by the electrophilic α,β-unsaturated carbonyl group in its cyclopentenone ring. Indeed, cyclopentenone ring-containing molecules are known to covalently interact with cysteine residues present in different proteins [36]. To assess molecular mechanisms by which 15d-PMJ2 elicits antitumor activity in skin cancer cells, we synthesized an analog of 15d-PMJ2 (neutral-PMJ2; Figure 6A) that lacks the reactive double bond. In B16F10 and JWF2 cells, neutral-15d-PMJ2 did not increase caspase-3/7 activity compared to control (Figure 6B, Supplementary Figure 3A). This was in stark contrast to 15d-PMJ2 which increased caspase-3/7 activity by 768% and 372% in B16F10 and JWF2 cells, respectively. To determine the role of the reactive double bond in the generation of oxidative stress, JWF2 and B16F10 skin cancer cells were incubated with an oxidative-stress probe following treatment with neutral-15d-PMJ2 or 15d-PMJ2. Both 15d-PMJ2 and neutral-15d-PMJ2 caused a significant increase in DCFDA fluorescence compared to control, with higher levels in 15d-PMJ2 treated cells (Figure 6C, Supplementary Figure 3B). In addition, we assessed cytosolic and mitochondrial calcium levels and found that accumulation of Ca^2+^ in both intracellular compartments was dependent on the double bond (Figure 6D and 6E, Supplementary Figure 3C and 3D). Finally, we determined that B16F10 cells treated with neutral-PMJ_2_ did not undergo mPTP opening or exhibit decreased cellular respiration compared to 15d-PMJ_2_ (Figure 6F and 6G), a finding consistent with the absence of an apoptotic effect by neutral-15d-PMJ_2_ (Figure 6B). These results demonstrate the importance of the cyclopentenone moiety in PERK/IP_3_R/mPTP-dependent apoptosis by15d-PMJ_2_.

## DISCUSSION

**Figure 7:**
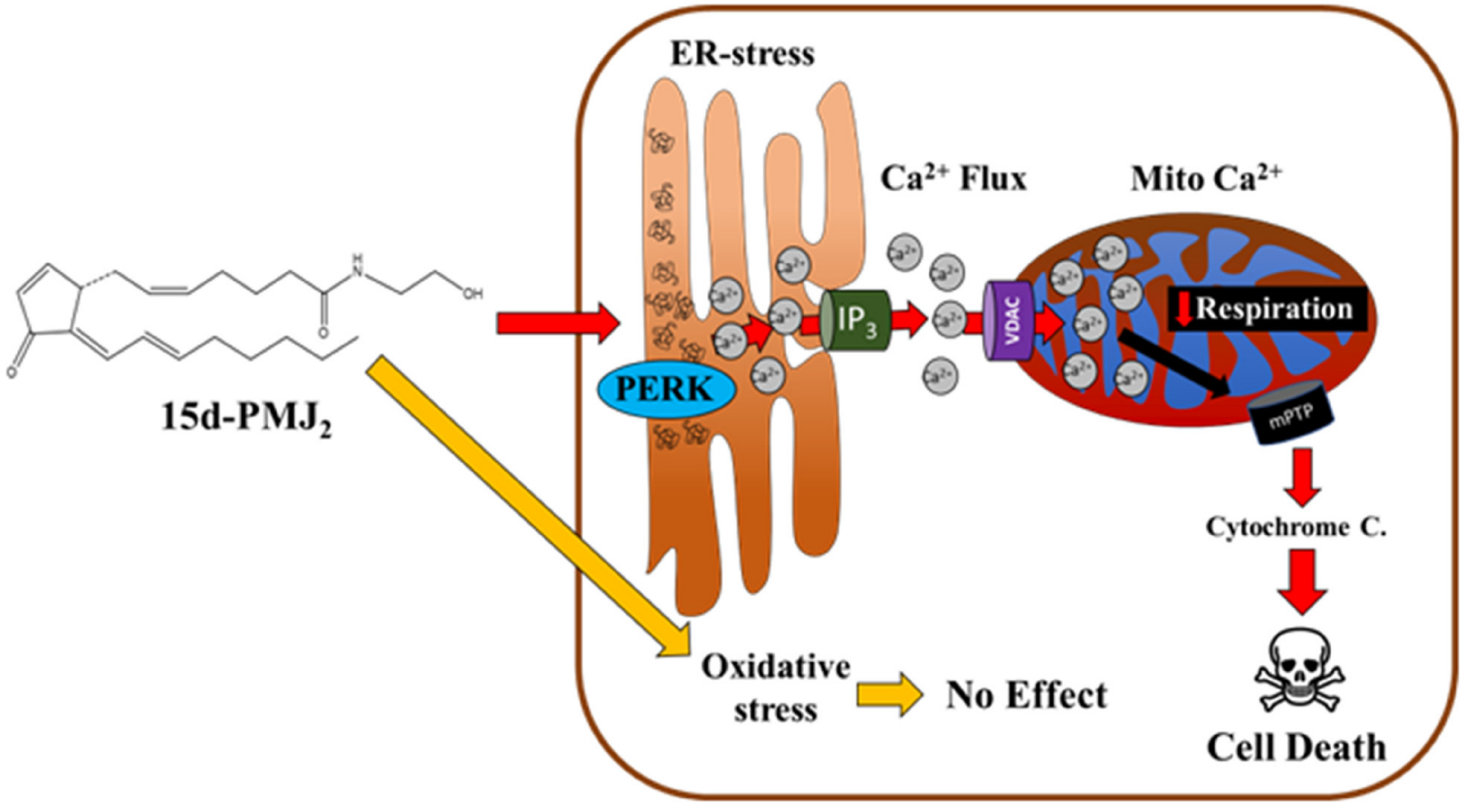
Proposed mechanism of 15d-PMJ_2_-mediated apoptosis. 15d-PMJ_2_ initiates ER stress-induced death primarily through the ER sensor, PERK. PERK activation causes Ca^2+^ translocation to the cytoplasm and mitochondria through ER-resident IP3R. Ca^2+^ flux to the mitochondria causes Ca^2+^ overload, mPTP opening, and mitochondrial cytochrome c loss, ultimately triggering cell death.

The goal of the present study was to determine the therapeutic potential of 15d-PMJ_2_ against melanoma by gaining insight into molecular mechanisms underlying its apoptotic activity. Our group previously found 15d-PMJ_2_ selectively induced ER stress and cell death in tumorigenic compared to non-tumorigenic skin cancer cell lines. Moreover, 15d-PMJ_2_ was effective at decreasing melanoma tumor growth *in vivo*. Building on these findings, the current study elucidated the role of the ER stress pathway and subsequent calcium flux in 15d-PMJ_2_-mediated tumor cell death (Figure 7). We found that in melanoma and cSCC cells treated with 15d-PMJ_2_, the ER stress sensor, PERK, was necessary for cell death. 15d-PMJ_2_-induced cytotoxicity also required the release of calcium from the ER through an ER stress-dependent pathway. ER stress generated by 15d-PMJ_2_ was determined to increase mitochondrial Ca^2+^ and facilitate the opening of the permeability transition pore thereby having a negative effect cellular respiration. Despite this, direct exposure of mitochondria to 15d-PMJ_2_ had no appreciable impact on bioenergetics. Moreover, the cyclopentenone double bond in 15d-PMJ_2_ was critical for the generation of ER stress, Ca^2+^ mobilization, mitochondrial permeability, and derangements in respiration. Taken together, these data suggest that an anti-melanoma mechanism of 15d-PMJ_2_ involves its double bond which causes PERK-dependent Ca^2+^ transit to the mitochondria and the subsequent release of cytochrome c.

In the presence of persistent or excessive ER stress, the apoptotic cascade is initiated by the ER stress sensors, PERK, ATF6, and IRE1 [37, 38]. To promote apoptosis, PERK increases the expression of the transcription factor, CHOP10. CHOP10 transactivates GADD34, a member of a protein complex that dephosphorylates eIF2a, thus allowing the expression of proapoptotic molecules. Through a similar pathway, the transcription factor, ATF6, triggers an apoptotic response by upregulating CHOP10. For IRE1, a key molecule involved in its apoptotic effect is TRAF2. IRE1:TRAF2 complexes catalyze the phosphorylation of Ask1 which activates JNK or p38, two MAP kinases that regulate the activity of pro- and anti-apoptotic proteins [39]. In addition, the endoribonuclease activity of IRE1 is suspected to play a role in apoptosis through regulated IRE1- dependent decay (RIDD) of mRNA molecules that encode anti-apoptotic proteins [37]. Although each of these ER stress sensors can drive the apoptotic response, PERK is the primary mediator of this outcome in most studies. For example, the inhibition of PERK prevented CHOP10 upregulation and the initiation of apoptosis in colon cancer cells treated with the dihydroartemisinin, NSC735847 [40]. In a different study, blockade of PERK inhibited apoptosis in colorectal cancer cells exposed to a derivative of the natural compound, curcumin [41]. Similar to these findings, we report ER stress-mediated apoptosis was propagated through PERK in 15d-PMJ_2_ treated cells as the blockade of PERK (GSK2606141) and inactivation of downstream eIF2α (salubrinal) prevented cell death. On the other hand, the inhibition of IRE1 or ATF6 did not suppress 15d-PMJ_2_ -induced cell death. Moreover, a previous study from our group revealed that PERK was required for ER stress, apoptosis, and the emission of damage associated molecular patterns (DAMPs), which caused dendritic cell maturation [42]. Hence, our collective findings shed light on the importance of PERK in 15d-PMJ_2_-mediated death and downstream signaling pathways.

Although we found PERK was required for apoptosis by 15d-PMJ_2_, Ca^2+^ also played a prominent role in its cytotoxic activity. Ca^2+^ initiates cell death through different molecular pathways [34]. For instance, cytosolic Ca^2+^ accumulation leads to activation of calpain proteases which cleave apoptosis-inducing factors such as poly-ADP ribose polymerase (PARP), caspase-3, and Bid to cause cell death [43]. It has also been demonstrated that IP_3_R-mediated Ca^2+^ transit to the mitochondria causes cell death through enhanced respiratory chain activity and generation of ROS [44]. In addition, excessive accumulation of Ca^2+^ within the mitochondria (a process referred to as Ca^2+^ overload), results in irreversible opening of the mPTP, causing the release of resident proapoptotic factors and activation of the apoptotic cascade [31]. The current study determined 15d-PMJ_2_ increased Ca^2+^ export from the ER via IP_3_R which led to an increase in mitochondrial Ca^2+^, mPTP opening, mitochondrial dysfunction, and cell death. In line with these results, IP3R channel inhibition rescued the mitochondria from respiratory failure which supported cell survival. Moreover, we observed that the Ca^2+^ regulated cytotoxicity of 15d-PMJ_2_ was mediated by PERK. While PERK-mediated Ca^2+^ accumulation in the mitochondria was critical for 15d-PMJ_2_ cell death, the route by which Ca^2+^ was transmitted to this organelle is unclear. Ca^2+^ flux to the mitochondria facilitates NADH formation, respiratory chain activity and ATP production while mitochondrial Ca^2+^ overload precipitates death. The mitochondria can import ER-derived Ca^2+^ from the cytoplasm or it can obtain Ca^2+^ directly from the ER. Ca^2+^ flow between the ER-mitochondrial interface occurs at specialized structures known as MAMs [7, 45]. Within MAMs, tethering proteins including the chaperone, glucose-regulated protein 75 (GRP75), anchor mitochondrial-VDACs and ER-IP_3_R to one another [45]. MAMs also contain ER stress-associated proteins such as IRE1 and PERK [7, 46, 47]. Given the importance of both PERK and IP_3_R in the formation of MAMs, the transfer of Ca^2+^ between the ER and mitochondria, and Ca^2+^ initiated death by 15d-PMJ_2_, our future studies will examine how these pathways intersect to regulate cell fate.

Several groups have reported that cyclopentenone prostaglandins including arachidonic acid-derived, 15-deoxy-Δ^12,14^-prostaglandin J_2_, directly interact with the mitochondria and diminish its activity [18, 35, 48]. Specifically, Martinez et. al., found that 15-deoxy-Δ^12,14^-prostaglandin J_2_ is a potent inhibitor of complex 1 which was reversed in the presence of the reducing agent DTT [18]. In addition, 15-deoxy-Δ^12,14^-prostaglandin J_2_ caused a significant reduction in complex 1 respiration and oxygen consumption through a calcium-dependent process [18, 19]. Therefore, an important goal of the current study was to determine whether the cyclopentenone prostamide, 15d-PMJ_2_, also caused mitochondrial impairment. According to our data, 15d-PMJ_2_ increased mPTP permeability and it decreased mitochondrial respiration in intact cells. This disruption in mitochondrial function was reversed by the mPTP inhibitor, cyclosporin A, suggesting that substrates and cofactors were lost through the mPTP. However, in isolated mitochondria, 15d-PMJ_2_ had no effect on mPTP opening, respiration, or other facets of mitochondrial bioenergetics. These results contradict the aforementioned studies with 15-deoxy-Δ^12,14^-prostaglandin J_2_ and suggest that the structural differences between these molecules may have an impact on their activity. Supporting this idea, Matias et al., found that amide-containing prostaglandins (prostamides) had different receptor binding activity and biological functions than their carboxyl-containing prostaglandin counterparts [49]. Furthermore, the prostaglandins, PGE_2_ and PGD_2_, have the same chemical formula but these molecules differ in the orientation of their 3-hydroxy-cyclopentanone ring. Despite this subtle difference, PGE_2_ and PGD_2_ bind to unique receptors and have opposing activities. Hence, it is plausible that the structurally similar molecules, 15d-PMJ_2_ and 15-deoxy-Δ^12,14^-prostaglandin J_2_, cause mitochondrial dysfunction through distinct mechanisms.

Another important moiety in the chemical structure of 15d-PMJ_2_ is the electrophilic α,β-unsaturated carbonyl group on its cyclopentenone ring that readily reacts with thiol groups of cellular proteins [50]. The same electrophilic double bond is present in all cyclopentenone prostaglandins including the J- and A-series prostaglandins and prostamides. To determine the significance of the double bond, we synthesized a neutral analog of 15d-PMJ_2_, which lacks the double bond, using the same approach described by Ladin et al. [25] Elimination of the reactive group in 15d-PMJ_2_ prevented apoptotic cell death in melanoma and NMSC cells. Moreover, the double bond was needed to increase cytoplasmic/mitochondrial Ca^2+^, induce mPTP opening, and suppress mitochondrial respiration. In contrast, the double bond was not required for ROS generation and ROS was not needed for 15d-PMJ_2_-induced death because neutralizing ROS did not prevent its cytotoxicity. This validates that the double bond is a critical determinant of 15d-PMJ_2_ cytotoxicity. However, the specific proteins to which 15d-PMJ_2_ interacts to initiate this lethal event have not been identified. In cells treated with arachidonic acid derived 15-deoxy-Δ^12,14^-prostaglandin J_2_ the lethal effect of this molecule has been attributed to the interaction of the double bond with glutathione, mitochondria respiratory complex 1, or antioxidant regulator Keap1 [18, 50, 51]. Based upon these findings, our group is seeking to identify cellular proteins that form adducts with 15d-PMJ_2_ to initiate cell death. The detection of these interacting proteins will facilitate biomarker discovery and optimization of 15d-PMJ_2_ activity.

In the present study, we investigated the molecular mechanism of 15d-PMJ_2_ in melanoma and found that this prostamide exerts its anti-tumor activity through the generation of ER stress (Figure 7). Activation of ER stress caused an increase in cytoplasmic Ca^2+^ levels through ER-resident IP_3_R. This ER stress-mediated calcium mobilization triggered the translocation of Ca^2+^ to the mitochondria followed by an increase in permeability of the mPTP and mitochondrial respiration impairment. Elimination of the electrophilic double bond in 15d-PMJ_2_ abolished its activity implicating the cyclopentenone moiety as a pharmacophore of this compound.

## MATERIALS AND METHODS

### Antibodies and reagents

15-deoxy, Δ^12,14^-prostamide J_2_ and 9,10-dihydro-prostamide J_2_ were synthesized, and their identity and purity were verified according to our previously published methods [25]. GSK2606414, STF083, Ceapin-A7, salubrinal, thapsigargin, 4-phenylbutyric acid, 2-aminoethoxydiphenyl borate, cyclosporin A and ruthenium red were purchased from Sigma-Aldrich (St. Louis, MO, USA). Caspase-Glo^®^ 3/7 and MTS reagent were from Promega Life Sciences (Madison, WI, USA).

### Cell lines and cell culture

The murine melanoma cell line B16F10 (a kind gift from Dr. Li Yang, East Carolina University) was cultured in Dulbecco’s Modified Eagle Medium (Invitrogen, Carlsbad, CA, USA) containing 10% heat-inactivated fetal bovine serum (FBS), penicillin (100 mg/mL), streptomycin (100 mg/mL), sodium pyruvate, and glutamine. The murine cutaneous squamous cell carcinoma (cSCC) cell line, JWF2 [a kind gift from Dr. Susan Fischer (University of Texas, MD Anderson Cancer Center, Smithville, TX, USA), was cultured in Eagle’s Minimal Essential Medium (US Biological, Swampscott, MS, USA) and contained 5% heat-inactivated FBS, penicillin (100 mg/mL), streptomycin (100 mg/mL), non-essential amino acids and glutamine.

### Cell viability assays

B16F10 and JWF2 cells were cultured in 96-well plates and incubated for 48 hours. The cells were treated with the appropriate agent and incubated for 12–24 hours. MTS reagent was added to each well and absorbance was measured at 495 nm on a Tecan Infinite Pro M200 plate reader (Mannedorf, Switzerland) as directed by the manufacturer (Promega, Madison, WI, USA).

### Caspase-3/7 activity assay

Cells were plated in white-walled 96-well plates and cultured for 48 hours. Serum-free medium containing the appropriate agents was added to the wells and the cells were incubated for the indicated amount of time. Caspase-Glo 3/7 reagent was then added to each well as directed by the manufacturer and luminescence was measured.

### Assessment of the cytosolic oxidative environment

The relative presence of molecules able to oxidize H_2_DCFDA, an indication of the presence of a cytosolic oxidative environment, was measured in JWF2 and B16F10 cells using the probe chloromethyl-2,7 dichlorodihydrofluorescein diacetate (CM-H2DCFDA; Molecular Probes, Invitrogen). Cells were treated with the appropriate agents using phenol red-free, serum-free medium for the indicated time followed by incubation with PBS supplemented with magnesium and calcium containing CM-H2DCFDA. Cells were then dissociated from the culture dish using Hank’s Enzyme-free dissociation buffer and resuspended in phenol red-free, serum-containing culture medium. Mean fluorescence was measured using an Accuri C6 flow cytometer (BD Accuri Cytometers, Ann Arbor, MI, USA) at an excitation wavelength of 488 nm and an emission filter of 533 ± 33 nm.

### Cytoplasmic Ca^2+^ measurements

To measure cytoplasmic Ca^2+^, treated cells were incubated with Fluo-4-NW at 37°C for 30 minutes in HBSS (without Mg^2+^ or Ca^2+^). Mean fluorescence was measured using a Tecan plate reader at an excitation wavelength of 493 nm and an emission filter of 513 ± 33 nm.

### Mitochondrial Ca^2+^ measurements

To measure mitochondrial Ca^2+^ levels, treated cells were incubated with Rhod-2 (1 mM) at 37°C for 30 minutes in PBS (without Mg^2+^ or Ca^2+^). Mean fluorescence was measured using an Accuri C6 flow cytometer at an excitation wavelength of 533 nm and an emission filter of 614 ± 33 nm.

### Evaluation of mitochondrial inner membrane (MIM) permeabilization

Calcein-AM enters cells and has access to every compartment including mitochondria. Cobalt is known to quench calcein fluorescence, but because cobalt does not freely enter mitochondria, calcein-AM fluorescence within the mitochondria is not quenched unless there is an increase in membrane permeability [52]. Briefly, cells were incubated with calcein-AM at a final concentration of 0.1 μM in DPBS for 30 min followed by 200 μM CoCl_2_ for 15 min. Calcein-AM fluorescence was measured using an excitation wavelength of 488 and a 535 ± 30-nm emission filter. The percentage of cells experiencing MIM permeabilization, meaning that the high calcein fluorescence was quenched by cobalt, was determined using the histogram plots of calcein-AM.

For experiments involving permeabilized cells, B16F10 cells were cultured for 12 hours in either vehicle control or 5 μM 15d-PMJ_2_. At the end of the 12-hour exposure, cells were washed in PBS and detached using 0.25% trypsin/EDTA. Following centrifugation at 300 × *g* for 7 minutes at room temperature, cell pellets were suspended in respiratory buffer supplemented with creatine (105 mM MES potassium salt, 30 mM KCl, 8 mM NaCl, 1 mM EGTA, 10 mM KH_2_PO_4_, 5 mM MgCl_2_, 0.25% BSA, 5 mM creatine monohydrate, pH 7.2). High-resolution respirometry measurements were performed using the Oroboros Oxgraph-2k (O2k; Oroboros Instruments, Innsbruck, Austria) in a 1.0 mL reaction volume at 37°C. Following an assessment of basal respiration, cells were permeabilized with digitonin (0.02 mg/mL), and respiratory flux was measured in response to the following additions: a) pyruvate, malate, glutamate, octanoyl-carnitine, succinate (P/M/G/S/O, 5 mM/1 mM/5 mM/0.2 mM/5 mM); b) ATP free energy (−54.16 kJ/mol); c) cytochrome C (0.01 mM). Data were normalized to viable cell count.

### Mitochondrial isolation and enzymatic characterization

Differential centrifugation was employed to prepare isolated mitochondria from B16F10 cells, as previously described [53]. The buffers used for all isolations were as follows: buffer A-MOPS (50 mM; pH = 7.1), KCl (100 mM), EGTA (1 mM), MgSO4 (5 mM); buffer B-buffer A, supplemented with bovine serum albumin (BSA; 2 g/L). For each isolation, cells were washed in PBS and detached using 0.25% trypsin/EDTA. Following centrifugation at 300 × *g* for 7 minutes at room temperature, cell pellets were resuspended in ice-cold buffer B, and the cells were homogenized via a Teflon pestle and borosilicate glass vessel for 40 passes. The homogenate was centrifuged at 800 × *g* for 10 min at 4°C. The supernatant was then pipetted into a separate tube, and the pellet was again resuspended in buffer B, homogenized, and centrifuged at 800 × *g*. This process was repeated a total of 3 times, and the supernatants from each of the 3 rounds of homogenization were pooled together and centrifuged at 10,000 × *g* for 10 min at 4°C. The mitochondrial pellet was then washed in buffer A, transferred to a microcentrifuge tube, and centrifuged again at 10,000 × *g* for 10 min at 4°C. Buffer A was aspirated from each tube, and final mitochondrial pellets were suspended in buffer A. Protein content was determined via the Pierce BCA protein assay. Respiratory kinetics were assessed at 37°C, as described above, using isolated mitochondria. Drug exposures were performed directly in the cuvette or well plate with either vehicle (0.1% DMSO) or 15d-PMJ_2_ (5 μM). Mitochondrial respiration was assessed in mitochondrial alone (mt), as well as in response to energization with glutamate/malate (G/M), octanoyl/malate (Oct/M), or succinate/rotenone (S/R). Respiratory kinetics were then assessed across a span of ATP free energies (ΔG_ATP_) using the creatine kinase energetic clamp [53, 54]. Oligomycin (Oligo) was added to inhibit ATP synthase, followed by titration with a respiratory uncoupler (FCCP). Respiration data were normalized to citrate synthase (CS) activity and expressed in pmol/s/CS. Individual activities of various matrix enzymes were assessed in a 96-well plate using alamethicin (0.03 mg/mL) permeabilized mitochondria, as previously described [55].

### Cellular respiration assay

3,000 B16F10 melanoma cells in medium without growth factors were seeded to each well of Seahorse XFe96 plates (Seahorse Bioscience, MA, USA) and grown for 48 hours at 37°C before treatment with various inhibitors, Neutral-PMJ_2_ and/or 15d-PMJ_2_. The cells were incubated with inhibitors for 30 mins in phenol red-free DMEM medium without fetal bovine serum. Following incubation with inhibitors, appropriate concentrations of 15d-PMJ_2_ were added to cells. Basal cellular respiration was measured on a Seahorse XFe96 flux analyzer according to manufacturer’s protocol. Each of the three independent experiments was performed in triplicate.

### Statistical analysis

Data are representative of three independent experiments unless otherwise indicated. Data are presented as mean ± standard error of the mean (SEM). Student’s *t*-test, one- or two-way ANOVA followed by Tukey’s post-hoc analysis was carried out as appropriate using GraphPad Prism and Microsoft Excel.

## SUPPLEMENTARY MATERIALS



## Abbreviations

2-APB: 2-Aminoethoxydiphenyl borate
15d-PGJ 2: 15-deoxy-Δ 12,14 -prostaglandin J 2
15d-PMJ 2: 15-deoxy-Δ 12,14 -prostamide J 2
4-PBA: 4-phenylbutrate
AA: Arachidonic acid
AEA: Arachidonoyl-ethanolamide (also known as anandamide)
ANOVA: Analysis of Variance
ATF: Activated transcription factor
BAP31: B-cell receptor associated protein 31
Bcl-2: B-cell lymphoma 2
BiP/GRP78: Binding immunoglobulin protein/78kDa glucose-regulated protein
CHOP10: C/EBP homologous protein
CM-H 2: DCFDA Chloromethyl-2’, 7’ dichlorodihydrofluorescein diacetate
DAMP: Damage associated molecular patterns
DMSO: Dimethyl sulfoxide
eIF2α: Eukaryotic initiation factor -2 alpha
ER: Endoplasmic reticulum
ERO1α: ER oxidoreductase 1 alpha
FBS: Fetal bovine serum
GSH: Glutathione
IP 3 R: Inositol 1,4,5-triphosphate receptor
IRE1: Inositol requiring kinase-1
MAM: Mitochondrial-associated ER membrane
MCU: Mitochondrial calcium uniporter
mPTP: Mitochondrial permeability transition pore
MTS: (3-(4,5-dimethylthiazol-2-yl)-5-(3carboxymethoxyphenyl)-2-(4-Sulfophenyl)-2H tetrazolium
NAC: N-acetyl cysteine
NMSC: Non-melanoma skin cancer
PARP: Poly (ADP-ribose) polymerase
PBS: Phosphate buffered saline
PERK: Double-stranded RNA-activated protein kinase (PKR)-like endoplasmic reticulum kinase
PG: Prostaglandin
PGJ 2: Prostaglandin J 2
PM: Prostamides
PMD 2: Prostamide D 2
PPAR: Peroxisome proliferator-activated receptor
P-PERK: Phosphorylated PERK
ROS: Reactive oxygen species
RR: Ruthenium Red
RyR: Ryanodine receptor
SCC: Squamous cell carcinoma
SERCA: Sarco/endoplasmic reticulum Ca^2+^-ATPase
SEM: Standard error of the mean
Sig-1R: Sigma-1 receptor
UPR: Unfolded protein response
VDAC: Voltage-dependent anion-selective channel
XBP-1: X box-binding protein

## References

[1] Siegel RL , Miller KD , Fuchs HE , Jemal A . Cancer statistics, 2022. CA Cancer J Clin. 2022; 72:7–33. 10.3322/caac.21708. 35020204

[2] Kim C , Kim B . Anti-Cancer Natural Products and Their Bioactive Compounds Inducing ER Stress-Mediated Apoptosis: A Review. Nutrients. 2018; 10:1021. 10.3390/nu10081021. 30081573 PMC6115829

[3] Kaneko M , Imaizumi K , Saito A , Kanemoto S , Asada R , Matsuhisa K , Ohtake Y . ER Stress and Disease: Toward Prevention and Treatment. Biol Pharm Bull. 2017; 40:1337–43. 10.1248/bpb.b17-00342. 28867719

[4] Tabas I , Ron D . Integrating the mechanisms of apoptosis induced by endoplasmic reticulum stress. Nat Cell Biol. 2011; 13:184–90. 10.1038/ncb0311-184. 21364565 PMC3107571

[5] Malhotra JD , Kaufman RJ . Endoplasmic reticulum stress and oxidative stress: a vicious cycle or a double-edged sword? Antioxid Redox Signal. 2007; 9:2277–93. 10.1089/ars.2007.1782. 17979528

[6] Sano R , Reed JC . ER stress-induced cell death mechanisms. Biochim Biophys Acta. 2013; 1833:3460–70. 10.1016/j.bbamcr.2013.06.028. 23850759 PMC3834229

[7] Verfaillie T , Rubio N , Garg AD , Bultynck G , Rizzuto R , Decuypere JP , Piette J , Linehan C , Gupta S , Samali A , Agostinis P . PERK is required at the ER-mitochondrial contact sites to convey apoptosis after ROS-based ER stress. Cell Death Differ. 2012; 19:1880–91. 10.1038/cdd.2012.74. 22705852 PMC3469056

[8] Clarke HJ , Chambers JE , Liniker E , Marciniak SJ . Endoplasmic reticulum stress in malignancy. Cancer Cell. 2014; 25:563–73. 10.1016/j.ccr.2014.03.015. 24823636

[9] Li G , Mongillo M , Chin KT , Harding H , Ron D , Marks AR , Tabas I . Role of ERO1-alpha-mediated stimulation of inositol 1,4,5-triphosphate receptor activity in endoplasmic reticulum stress-induced apoptosis. J Cell Biol. 2009; 186:783–92. 10.1083/jcb.200904060. 19752026 PMC2753154

[10] Hayashi T , Su TP . Sigma-1 receptor chaperones at the ER-mitochondrion interface regulate Ca(2+) signaling and cell survival. Cell. 2007; 131:596–610. 10.1016/j.cell.2007.08.036. 17981125

[11] Breckenridge DG , Stojanovic M , Marcellus RC , Shore GC . Caspase cleavage product of BAP31 induces mitochondrial fission through endoplasmic reticulum calcium signals, enhancing cytochrome c release to the cytosol. J Cell Biol. 2003; 160:1115–27. 10.1083/jcb.200212059. 12668660 PMC2172754

[12] Roy SS , Hajnóczky G . Calcium, mitochondria and apoptosis studied by fluorescence measurements. Methods. 2008; 46:213–23. 10.1016/j.ymeth.2008.09.024. 18948203 PMC3799833

[13] Shin SW , Seo CY , Han H , Han JY , Jeong JS , Kwak JY , Park JI . 15d-PGJ2 induces apoptosis by reactive oxygen species-mediated inactivation of Akt in leukemia and colorectal cancer cells and shows *in vivo* antitumor activity. Clin Cancer Res. 2009; 15:5414–25. 10.1158/1078-0432.CCR-08-3101. 19690198

[14] Trindade-da-Silva CA , Reis CF , Vecchi L , Napimoga MH , Sperandio M , Matias Colombo BF , Alves PT , Ward LS , Ueira-Vieira C , Goulart LR . 15-Deoxy-Δ(12,14)-prostaglandin J2 Induces Apoptosis and Upregulates SOCS3 in Human Thyroid Cancer Cells. PPAR Res. 2016; 2016:4106297. 10.1155/2016/4106297. 27190500 PMC4852108

[15] Kuc C , Jenkins A , Van Dross RT . Arachidonoyl ethanolamide (AEA)-induced apoptosis is mediated by J-series prostaglandins and is enhanced by fatty acid amide hydrolase (FAAH) blockade. Mol Carcinog. 2012; 51:139–49. 10.1002/mc.20770. 21432910 PMC3134573

[16] Van Dross RT . Metabolism of anandamide by COX-2 is necessary for endocannabinoid-induced cell death in tumorigenic keratinocytes. Mol Carcinog. 2009; 48:724–32. 10.1002/mc.20515. 19148897

[17] Dionne S , Levy E , Levesque D , Seidman EG . PPARgamma ligand 15-deoxy-delta 12,14-prostaglandin J2 sensitizes human colon carcinoma cells to TWEAK-induced apoptosis. Anticancer Res. 2010; 30:157–66. 20150631

[18] Martínez B , Pérez-Castillo A , Santos A . The mitochondrial respiratory complex I is a target for 15-deoxy-delta12,14-prostaglandin J2 action. J Lipid Res. 2005; 46:736–43. 10.1194/jlr.M400392-JLR200. 15654126

[19] Landar A , Shiva S , Levonen AL , Oh JY , Zaragoza C , Johnson MS , Darley-Usmar VM . Induction of the permeability transition and cytochrome c release by 15-deoxy-Delta12,14-prostaglandin J2 in mitochondria. Biochem J. 2006; 394:185–95. 10.1042/BJ20051259. 16268779 PMC1386016

[20] Elhassanny AEM , Ladin DA , Soliman E , Albassam H , Morris A , Kobet R , Thayne K , Burns C , Danell AS , Van Dross R . Prostaglandin D_2_-ethanolamide induces skin cancer apoptosis by suppressing the activity of cellular antioxidants. Prostaglandins Other Lipid Mediat. 2019; 142:9–23. 10.1016/j.prostaglandins.2019.03.001. 30858059

[21] Soliman E , Van Dross R . Anandamide-induced endoplasmic reticulum stress and apoptosis are mediated by oxidative stress in non-melanoma skin cancer: Receptor-independent endocannabinoid signaling. Mol Carcinog. 2016; 55:1807–21. 10.1002/mc.22429. 26513129

[22] Soliman E , Henderson KL , Danell AS , Van Dross R . Arachidonoyl-ethanolamide activates endoplasmic reticulum stress-apoptosis in tumorigenic keratinocytes: Role of cyclooxygenase-2 and novel J-series prostamides. Mol Carcinog. 2016; 55:117–30. 10.1002/mc.22257. 25557612

[23] Fišar Z , Singh N , Hroudová J . Cannabinoid-induced changes in respiration of brain mitochondria. Toxicol Lett. 2014; 231:62–71. 10.1016/j.toxlet.2014.09.002. 25195527

[24] Catanzaro G , Rapino C , Oddi S , Maccarrone M . Anandamide increases swelling and reduces calcium sensitivity of mitochondria. Biochem Biophys Res Commun. 2009; 388:439–42. 10.1016/j.bbrc.2009.08.037. 19679102

[25] Ladin DA , Soliman E , Escobedo R , Fitzgerald TL , Yang LV , Burns C , Van Dross R . Synthesis and Evaluation of the Novel Prostamide, 15-Deoxy, Δ^12,14^-Prostamide J_2_, as a Selective Antitumor Therapeutic. Mol Cancer Ther. 2017; 16:838–49. 10.1158/1535-7163.MCT-16-0484. 28292936

[26] Chen YC , Shen SC , Tsai SH . Prostaglandin D(2) and J(2) induce apoptosis in human leukemia cells via activation of the caspase 3 cascade and production of reactive oxygen species. Biochim Biophys Acta. 2005; 1743:291–304. 10.1016/j.bbamcr.2004.10.016. 15843042

[27] Chen K , Dai W , Wang F , Xia Y , Li J , Li S , Liu T , Zhang R , Wang J , Lu W , Zhou Y , Yin Q , Zheng Y , et al. Inhibitive effects of 15-deoxy-Δ(12),(14)-prostaglandin J2 on hepatoma-cell proliferation through reactive oxygen species-mediated apoptosis. Onco Targets Ther. 2015; 8:3585–93. 10.2147/OTT.S92832. 26664142 PMC4671813

[28] Oparka M , Walczak J , Malinska D , van Oppen LMP , Szczepanowska J , Koopman WJH , Wieckowski MR . Quantifying ROS levels using CM-H_2_DCFDA and HyPer. Methods. 2016; 109:3–11. 10.1016/j.ymeth.2016.06.008. 27302663

[29] Saito K , Matsuoka Y , Yamada KI . Reaction targets of antioxidants in azo-initiator or lipid hydroperoxide induced lipid peroxidation. Free Radic Res. 2020; 54:301–10. 10.1080/10715762.2020.1761020. 32338088

[30] Pinton P , Giorgi C , Siviero R , Zecchini E , Rizzuto R . Calcium and apoptosis: ER-mitochondria Ca2^+^ transfer in the control of apoptosis. Oncogene. 2008; 27:6407–18. 10.1038/onc.2008.308. 18955969 PMC2844952

[31] Marchi S , Patergnani S , Missiroli S , Morciano G , Rimessi A , Wieckowski MR , Giorgi C , Pinton P . Mitochondrial and endoplasmic reticulum calcium homeostasis and cell death. Cell Calcium. 2018; 69:62–72. 10.1016/j.ceca.2017.05.003. 28515000

[32] Luciani DS , Gwiazda KS , Yang TL , Kalynyak TB , Bychkivska Y , Frey MH , Jeffrey KD , Sampaio AV , Underhill TM , Johnson JD . Roles of IP3R and RyR Ca2^+^ channels in endoplasmic reticulum stress and beta-cell death. Diabetes. 2009; 58:422–32. 10.2337/db07-1762. 19033399 PMC2628616

[33] Liang SH , Zhang W , McGrath BC , Zhang P , Cavener DR . PERK (eIF2alpha kinase) is required to activate the stress-activated MAPKs and induce the expression of immediate-early genes upon disruption of ER calcium homoeostasis. Biochem J. 2006; 393:201–9. 10.1042/BJ20050374. 16124869 PMC1383678

[34] Krebs J , Agellon LB , Michalak M . Ca(2+) homeostasis and endoplasmic reticulum (ER) stress: An integrated view of calcium signaling. Biochem Biophys Res Commun. 2015; 460:114–21. 10.1016/j.bbrc.2015.02.004. 25998740

[35] Ceaser EK , Ramachandran A , Levonen AL , Darley-Usmar VM . Oxidized low-density lipoprotein and 15-deoxy-delta 12,14-PGJ2 increase mitochondrial complex I activity in endothelial cells. Am J Physiol Heart Circ Physiol. 2003; 285:H2298–308. 10.1152/ajpheart.00508.2003. 12881207

[36] Shibata T . 15-Deoxy-Δ¹²,¹^4^-prostaglandin J_2_ as an electrophilic mediator. Biosci Biotechnol Biochem. 2015; 79:1044–49. 10.1080/09168451.2015.1012149. 26011133

[37] Iurlaro R , Muñoz-Pinedo C . Cell death induced by endoplasmic reticulum stress. FEBS J. 2016; 283:2640–52. 10.1111/febs.13598. 26587781

[38] Adams CJ , Kopp MC , Larburu N , Nowak PR , Ali MMU . Structure and Molecular Mechanism of ER Stress Signaling by the Unfolded Protein Response Signal Activator IRE1. Front Mol Biosci. 2019; 6:11. 10.3389/fmolb.2019.00011. 30931312 PMC6423427

[39] Mhaidat NM , Thorne R , Zhang XD , Hersey P . Involvement of endoplasmic reticulum stress in Docetaxel-induced JNK-dependent apoptosis of human melanoma. Apoptosis. 2008; 13:1505–12. 10.1007/s10495-008-0276-8. 18989785

[40] Elhassanny AEM , Soliman E , Marie M , McGuire P , Gul W , ElSohly M , Van Dross R . Heme-Dependent ER Stress Apoptosis: A Mechanism for the Selective Toxicity of the Dihydroartemisinin, NSC735847, in Colorectal Cancer Cells. Front Oncol. 2020; 10:965. 10.3389/fonc.2020.00965. 32626657 PMC7313430

[41] Wang H , Xu Y , Sun J , Sui Z . The Novel Curcumin Derivative 1g Induces Mitochondrial and ER-Stress-Dependent Apoptosis in Colon Cancer Cells by Induction of ROS Production. Front Oncol. 2021; 11:644197. 10.3389/fonc.2021.644197. 34195069 PMC8236884

[42] Elhassanny A , Escobedo R , Ladin D , Burns C , Van Dross R . Damage-associated molecular pattern (DAMP) activation in melanoma: investigation of the immunogenic activity of 15-deoxy, Δ^12,14^ prostamide J_2_. Oncotarget. 2020; 11:4788–802. 10.18632/oncotarget.27856. 33447347 PMC7779254

[43] Smith MA , Schnellmann RG . Calpains, mitochondria, and apoptosis. Cardiovasc Res. 2012; 96:32–37. 10.1093/cvr/cvs163. 22581845 PMC3444233

[44] Giorgi C , Marchi S , Pinton P . The machineries, regulation and cellular functions of mitochondrial calcium. Nat Rev Mol Cell Biol. 2018; 19:713–30. 10.1038/s41580-018-0052-8. 30143745

[45] Fujimoto M , Hayashi T . New insights into the role of mitochondria-associated endoplasmic reticulum membrane. Int Rev Cell Mol Biol. 2011; 292:73–117. 10.1016/B978-0-12-386033-0.00002-5. 22078959

[46] Cao Y , Chen Z , Hu J , Feng J , Zhu Z , Fan Y , Lin Q , Ding G . Mfn2 Regulates High Glucose-Induced MAMs Dysfunction and Apoptosis in Podocytes via PERK Pathway. Front Cell Dev Biol. 2021; 9:769213. 10.3389/fcell.2021.769213. 34988075 PMC8721005

[47] van Vliet AR , Agostinis P . When under pressure, get closer: PERKing up membrane contact sites during ER stress. Biochem Soc Trans. 2016; 44:499–504. 10.1042/BST20150272. 27068961

[48] Diers AR , Higdon AN , Ricart KC , Johnson MS , Agarwal A , Kalyanaraman B , Landar A , Darley-Usmar VM . Mitochondrial targeting of the electrophilic lipid 15-deoxy-Delta12,14-prostaglandin J2 increases apoptotic efficacy via redox cell signalling mechanisms. Biochem J. 2010; 426:31–41. 10.1042/BJ20091293. 19916962 PMC3079364

[49] Matias I , Chen J , De Petrocellis L , Bisogno T , Ligresti A , Fezza F , Krauss AH , Shi L , Protzman CE , Li C , Liang Y , Nieves AL , Kedzie KM , et al. Prostaglandin ethanolamides (prostamides): *in vitro* pharmacology and metabolism. J Pharmacol Exp Ther. 2004; 309:745–57. 10.1124/jpet.103.061705. 14757851

[50] Uchida K , Shibata T . 15-Deoxy-Delta(12,14)-prostaglandin J2: an electrophilic trigger of cellular responses. Chem Res Toxicol. 2008; 21:138–44. 10.1021/tx700177j. 18052108

[51] Oh JY , Giles N , Landar A , Darley-Usmar V . Accumulation of 15-deoxy-delta(12,14)-prostaglandin J2 adduct formation with Keap1 over time: effects on potency for intracellular antioxidant defence induction. Biochem J. 2008; 411:297–306. 10.1042/bj20071189. 18237271 PMC2683789

[52] Petronilli V , Miotto G , Canton M , Brini M , Colonna R , Bernardi P , Di Lisa F . Transient and long-lasting openings of the mitochondrial permeability transition pore can be monitored directly in intact cells by changes in mitochondrial calcein fluorescence. Biophys J. 1999; 76:725–34. 10.1016/S0006-3495(99)77239-5. 9929477 PMC1300077

[53] Nelson MA , McLaughlin KL , Hagen JT , Coalson HS , Schmidt C , Kassai M , Kew KA , McClung JM , Neufer PD , Brophy P , Vohra NA , Liles D , Cabot MC , Fisher-Wellman KH . Intrinsic OXPHOS limitations underlie cellular bioenergetics in leukemia. Elife. 2021; 10:e63104. 10.7554/eLife.63104. 34132194 PMC8221809

[54] Fisher-Wellman KH , Davidson MT , Narowski TM , Lin CT , Koves TR , Muoio DM . Mitochondrial Diagnostics: A Multiplexed Assay Platform for Comprehensive Assessment of Mitochondrial Energy Fluxes. Cell Rep. 2018; 24:3593–606.e10. 10.1016/j.celrep.2018.08.091. 30257218 PMC6237617

[55] McLaughlin KL , Hagen JT , Coalson HS , Nelson MAM , Kew KA , Wooten AR , Fisher-Wellman KH . Novel approach to quantify mitochondrial content and intrinsic bioenergetic efficiency across organs. Sci Rep. 2020; 10:17599. 10.1038/s41598-020-74718-1. 33077793 PMC7572412

