# Supplementary material for: Calcium signaling induced by 15-deoxy-prostamide-J2 promotes cell death by activating PERK, IP3R, and the mitochondrial permeability transition pore
Source: Oncotarget. 2022 Dec 29;13:1380–96. doi: 10.18632/oncotarget.28334 (PMC9799328; doi:10.18632/oncotarget.28334)
Supplement: Supplementary file 1 [file oncotarget-13-28334-s001.pdf]

Calcium signaling induced by 15-deoxy-prostamide-J_2 promotes cell death by activating PERK, IP3R, and the mitochondrial permeability transition pore

SUPPLEMENTARY MATERIALS

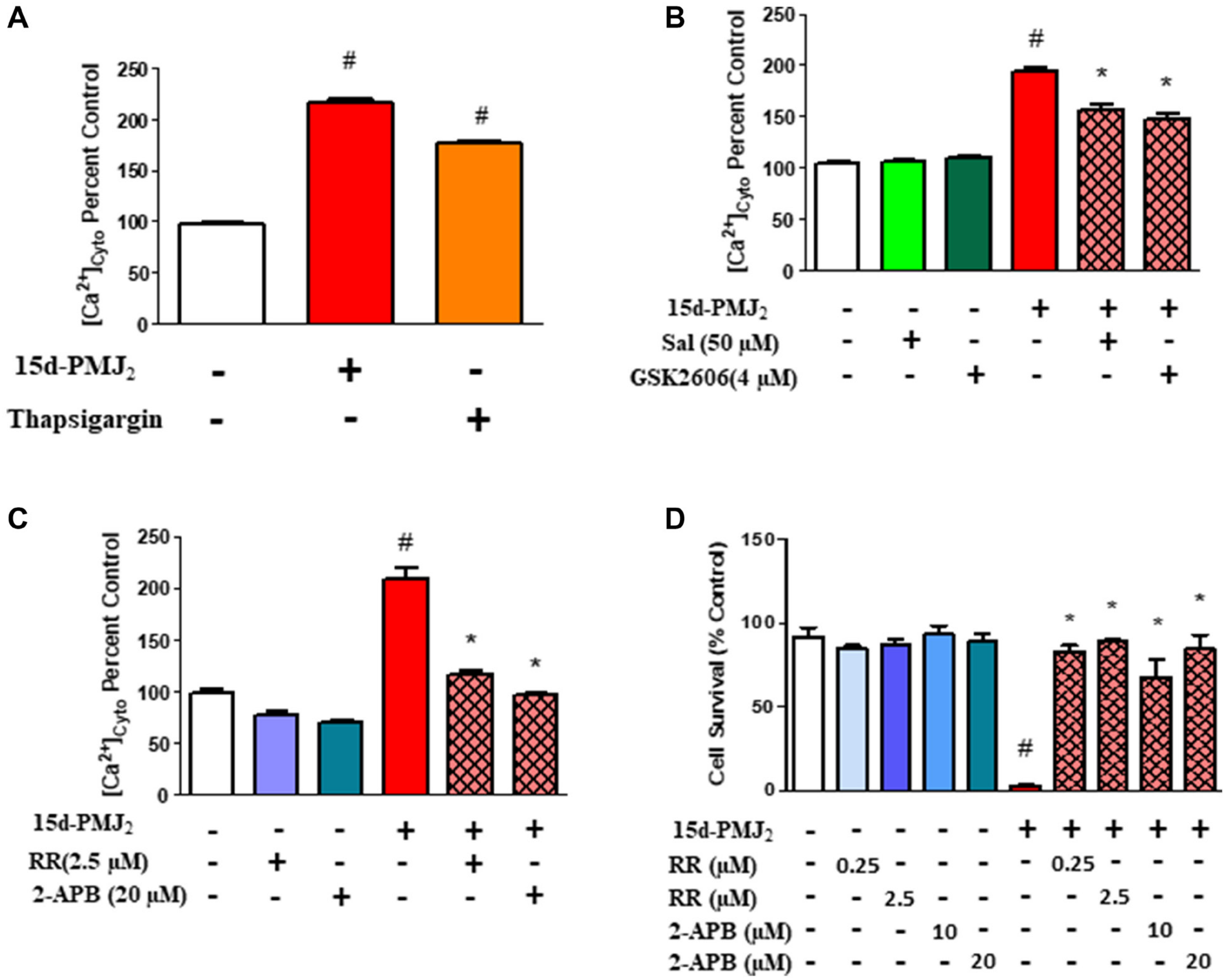

Supplementary Figure 1: Activation of Ca^{2+} channels by 15d-PMJ_2 is ER-stress mediated and required for death in cSCC, JWF2 cells. (**A**) JWF2 cells were treated with 5 μM 15d-PMJ_2, 10 μM thapsigargin or vehicle (culture medium containing 0.1% DMSO) for 1 hour. Cytoplasmic Ca^{2+} levels were measured by performing experiments with the Fluor-4 NW probe. (**B**) JWF2 cells were pretreated with salubrinal or GSK2606414 for 30 minutes, treated with 5 μM 15d-PMJ_2 or vehicle for 1 hour, and then cytoplasmic Ca^{2+} was detected. (**C**) JWF2 cells were pretreated with ruthenium red or 2-APB for 1 hour, treated with 5 μM 15d-PMJ_2 or vehicle for 1 hour, and then cytoplasmic Ca^{2+} levels were measured. (**D**) Cells were pretreated with RR (0.25 or 2.5 μM) or 2-APB (10 or 20 μM) for 1 hour followed by cell treatment with 15d-PMJ_2 or vehicle for 12 hour. Cell viability was determined by performing MTS assays. Data are presented as mean ± SEM and represent three independent experiments. $^{*}P < 0.05$, when comparing samples to 15d-PMJ_2-treated cells, $^{\#}P < 0.05$, when comparing samples to vehicle-treated cells.

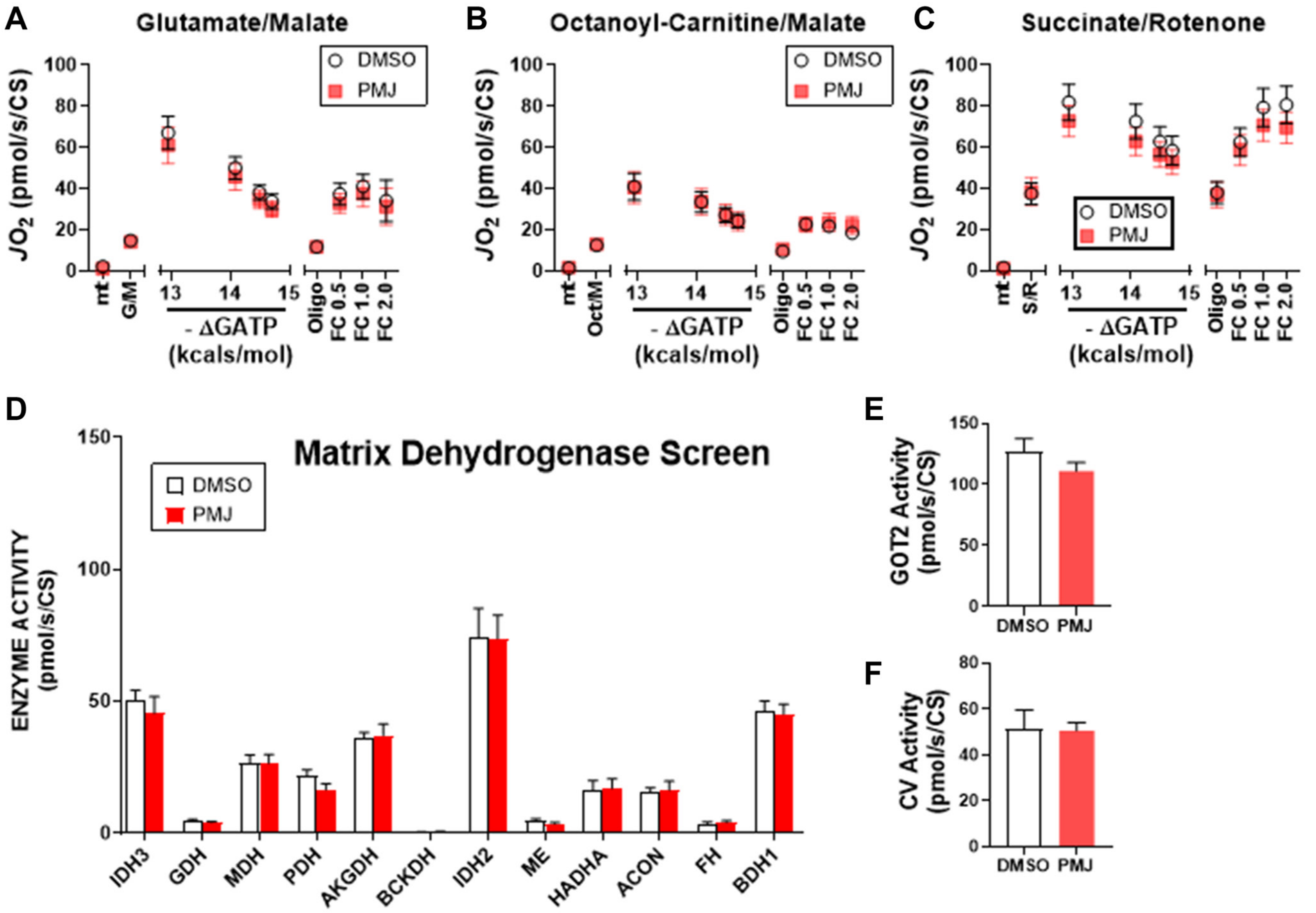

Supplementary Figure 2: Impact of 15d-PMJ$_2$ on mitochondrial bioenergetics. Isolated mitochondria were prepared from B16F10 cells. Isolated mitochondria were then treated directly in the cuvette or well plate with either 15d-PMJ$_2$ (5 μM) or vehicle (medium containing 0.1% DMSO). (**A**–**C**) Mitochondrial respiration was assessed in mitochondria alone (mt), as well as in response to energization with (A) glutamate/malate (G/M), (B) octanoyl-carnitine/malate (Oct/M), or (C) succinate/rotenone (S/R). Respiratory kinetics were then assessed across a span of ATP free energies (ΔGATP) using the creatine kinase energetic clamp. Oligomycin (Oligo) was added to inhibit ATP synthase, followed by titration with a respiratory uncoupler (FCCP). Respiration data was normalized to citrate synthase (CS) activity and is expressed in pmol/s/CS. (**D**–**F**) Individual activities of various matrix dehydrogenase enzymes was assessed. Data are expressed as pmol/s/CS. Enzyme activity of (E) GOT2 and (F) Complex V are shown. Data are mean ± SEM, N = 6–8/group.

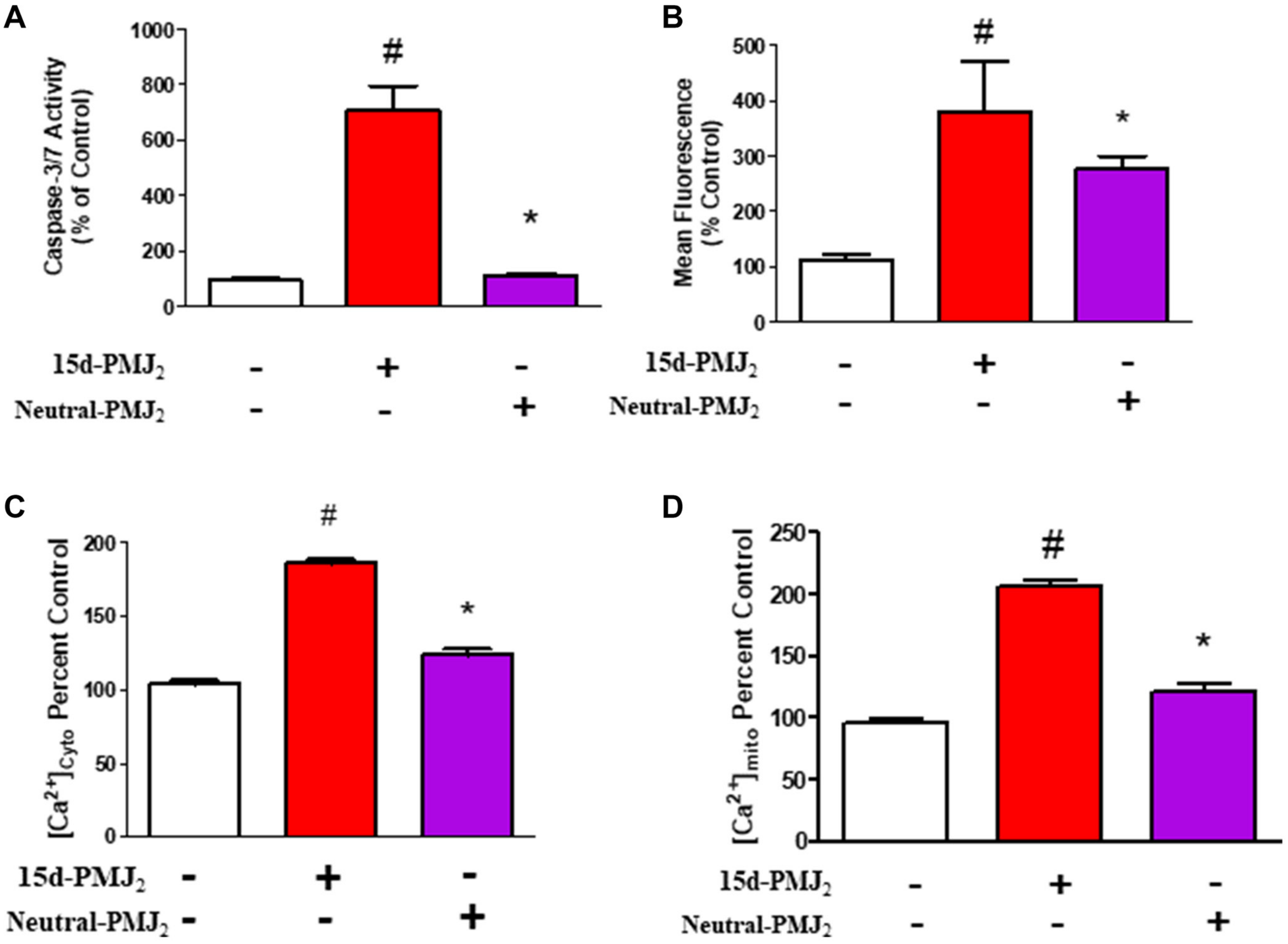

Supplementary Figure 3: Activity of 15d-PMJ$_2$ is mediated through the electrophilic double bond in cSCC cells. JWF2 cells were treated with 15d-PMJ$_2$, Neutral-15d-PMJ$_2$ or vehicle and then assayed for (**A**) Caspase-3/7 activity, (**B**) oxidative stress, (**C**) cytoplasmic Ca^{2+}, and (**D**) mitochondrial Ca^{2+}. Data represent mean ± SEM of three independent experiments and are expressed as percent of untreated group. $^{*}P < 0.05$, as compared to 15d-PMJ$_2$, $^{\#}P < 0.05$, as compared to vehicle.
